# ﻿A synopsis of the Campodeidae dipluran fauna from China (Arthropoda, Hexapoda) with a taxonomic key

**DOI:** 10.3897/zookeys.1239.144945

**Published:** 2025-05-23

**Authors:** Alberto Sendra, Jesús Selfa, Yun Bu, Yun-Xia Luan

**Affiliations:** 1 Colecciones Entomológicas Torres-Sala, Servei de Patrimoni Històric, Ajuntament de València, València, Spain; 2 Departament de Didàctica de les Ciències Experimentals i Socials, Facultat de Magisteri, Universitat de València, València, Spain; 3 Grup de recerca en Zoologia – ZOORECERC, Laboratori d’Investigació d’Entomologia, Departament de Zoologia, Universitat de València, Burjassot, València, Spain; 4 Natural History Research Center, Shanghai Natural History Museum, Shanghai Science & Technology Museum, Shanghai, China; 5 Guangdong Provincial Key Laboratory of Insect Development Biology and Applied Technology, Institute of Insect Science and Technology, School of Life Sciences, South China Normal University, Guangzhou, China

**Keywords:** Biodiversity, distribution, habitat, taxonomy, zoology

## Abstract

This study provides a detailed examination of the taxonomy and distribution of 25 described Campodeidae diplurans species from the Chinese fauna, which frequently inhabit soil and cave environments. It involves the revision and rewriting of the diagnoses and descriptions for all taxa, based on prepared samples from Chinese scientific collections and new, unprocessed specimens observed via scanning electron microscopy for three species (*Metriocampaurumqiensis*, *Leniwytsmaniaorientalis*, and *Lepidocampaweberi*). In addition, the first taxonomic key for Chinese Campodeidae species is presented. This biodiversity includes four subfamilies: Campodeinae (12 species), Plusiocampinae (7 species), Lepidocampinae (5 species), and Syncampinae (1 species). Among these, 16 species are endemic to China, including five genera exclusive to the region: *Pseudolibanocampa*, *Sinocampa*, and *Syncampa* in soil environments, and *Hubeicampa* and *Whittencampa* in caves. The subfamily Syncampinae is only known in China. The richness of Campodeidae in China is the highest in East Asia, but relatively lower compared to the well-sampled and studied Euro-Mediterranean region, located at the opposite end of the Palearctic region. Nevertheless, East Asia should be considered as the origin of three campodeid subfamilies: Plusiocampinae, Lepidocampinae, and Syncampinae.

## ﻿Introduction

Diplurans are one of three entognathous hexapod groups, in addition to proturans and collembolans, present in almost every soil, cave, or other empty subsurface space. Despite their ubiquity in terrestrial subsurface habitats, diplurans have been mostly overlooked in ecological studies and have not a solid worldwide revision since the monograph of campodeids by Condé (1956) and the Diplura checklist by Paclt (1957). As hexapods, diplurans have an insect-like body plan with three parts: head, thorax, and abdomen. The head has two frontal antennae, with all antennomeres equipped with their own set of muscles, and unique entognathous mouthparts partially hidden into two oral folds. The three thoracic segments lack wings (apterygote hexapods), and each has a pair of similar legs ending in a simple tarsus with two claws (pretarsus). The abdomen is divided into ten complete segments, some with vestiges of legs represented by a pair of articulated styli and eversible water-absorbing vesicles. The last abdominal segment bears the typically paired cerci, responsible for the common name two-pronged bristletails or ‘double tails’ that evolved into a variety of shapes and functions differing among families (Sendra et al. 2021a).

The biodiversity of diplurans is unequally distributed into ten families, which exhibit a large variety in body size and shape, behaviour, reproduction, and habitat preferences (Denis 1949; Koch 2001; Sendra 2015). Among these, Campodeidae (491 species) and Japygidae (343 species) are well represented in the world, followed by Parajapygidae (62), Evalljapygidae (47), and Projapygidae (42), and the remaining families (Anajapygidae, Dinjapygidae, Heterojapygidae, Octostigmatidae, and Procampodeidae) represent only 2% of the total diversity (Pagés 1959, 1989; Rusek 1982; Sendra 2021; Sendra et al. 2021a). In China, six families have been reported: Campodeidae, Japygidae, Parajapygidae, Heterojapygidae, Projapygidae, and Octostigmatidae (Yin 2000; Sendra and Deharveng 2020; Sendra et al. 2021b). In addition to these extant species another 14 fossil Diplura species have been described that reveal its interesting and primitive origins among the earliest geological evidence of hexapods, the beginning of the terrestrial animals (Kukalová−Peck 1987; Sánchez-García et al. 2023a, b; Wang et al. 2023).

So far, 25 species of Campodeidae distributed in China have been reported. The first and most significant contribution to the dipluran fauna of China was made by the Italian entomologist Filippo Silvestri, who in 1931 described or cited 13 species of China. Half a century later, Chinese entomologists took over in dipluran taxonomy. Io Chou and Tong Chen from the Northwest A&F University published four new species, including the description of the new genus *Sinocampa* in the subfamily Lepidocampinae (Chou and Chen 1980, 1981). A decade later, entomologists Rongdong Xie and Yiming Yang from the Shanghai Institute of Entomology worked intensely on dipluran taxonomy, providing the description of three new species and the proposal of two genera exclusive to China, *Pseudolibanocampa* and *Anisocampa* (Xie and Yang 1991).

Almost at the end of the last century, the French entomologist Bruno Condé published the first contribution on cave campodeids, thanks to the sampling effort of speleologists such as Josiane Lips. Condé (1993a) described the first troglobiont campodeid, a species with cave-adapted features, belonging to the subfamily Plusiocampinae. A few years later the American entomologist Lynn Ferguson reported a probable new species of the genus *Pacificampa* from a cave (Ferguson 1997). Only recently, two articles have reactivated the interest and importance of campodeids from China, both focused on cave-adapted species with the description of two new genera (*Hubeicampa* and *Whittencampa*) and one new species of *Pacificampa* (Sendra and Deharveng 2020; Sendra et al. 2021b).

The aim of this article is to provide a comprehensive synopsis of the campodeid dipluran fauna of China with a concise diagnosis for each taxon. These updates have allowed us to discuss the composition and distribution of this family in China in comparison with other regions worldwide, considering both soil and cave-dwelling species based on literature and collections deposited in several museums.

## ﻿Material and methods

We have reviewed collections from South China Normal University and Shanghai Entomological Museum CAS, using a Leica DMLS phase-contrast optical microscope for examination. A total of 21 specimens in alcohol of three Chinese species, *Metriocampaurumqiensis* Chou & Chen, 1980, *Leniwytsmaniaorientalis* (Silvestri, 1931), and *Lepidocampaweberi* Oudemans, 1890, were prepared for scanning electron microscopic (SEM) photography and sensilla measurements. Nine specimens were coated with palladium-gold and observed using a Hitachi S-4900 scanning electron microscope. Morphological descriptions and abbreviations follow Condé (1956). The term ‘gouge sensilla’ refers to the concavo-convex sensilla on the antennae (while the term ‘rosette-like’ refers to the epicuticle gland formation. To designate the position of macrosetae, we adopted the abbreviations of Condé (1955): ***ma***, medial-anterior; ***la***, lateral-anterior; ***lp***, lateral-posterior; ***mp***, medial-posterior; and ***post***, posterior macrosetae.

## ﻿Taxonomy

**Class Hexapoda Blainville, 1816**

**Order Diplura Börner, 1904**

**Suborder Rhabdura Cook, 1896**

**Family Campodeidae Lubbock, 1873**

### ﻿Subfamily Campodeinae

Taxon classificationAnimaliaDipluraCampodeidae

Condé, 1956: 94

2657DAF6-BCAC-5E07-B88B-38A24715F0FA

#### Diagnosis.

Epicuticle smooth or with microdenticles; presence of rosette-like formations; body covered with smooth to well-barbed macrosetae and setae; sensillum of third antennomere in dorsal or ventral position; labial pieces typical or slightly rotated in *Remycampa* ssp. and few *Podocampa* ssp.; pronotal macrosetae formula up to 1+1 *ma*, 1+1 *la*, 1+1 *lp*; 0 or 1 dorsal femoral macrosetae; tibia with 0–3 short ventral macrosetae; simple subequal claws without medial unguiculus except in *Eutrichocampahispanica* Silvestri, 1932; claws with lateral crests in *Litocampa* and *Haplocampa*; one pair of *ma* and *la* urotergal macrosetae at most and one or two pairs of lateral posterior urotergal macrosetae (rarely three).

#### Remarks.

Forty-three genera and a total of 388 species have been described (Condé 1956; Sendra et al. 2021a).

#### Distribution.

Although most of this subfamily is concentrated in the Nearctic and West Palearctic regions, several species have also been described in all realms of the Southern Hemisphere.

### ﻿Genus Campodea

Taxon classificationAnimaliaDipluraCampodeidae

Westwood, 1842

4B2A73B0-3E7D-5FAA-8940-027C99E48724

#### Diagnosis.

Notal macrosetae pattern as 1+1 *ma*, 1+1 *la*, 1+1 *lp* macrosetae on pronotum or more, and up to 1+1 *ma*, 1+1 *la*, 1+1 *lp* macrosetae on mesonotum and up to 1+1 *ma*, 1+1 *lp* on metanotum; without dorsal femoral macrosetae; with one short ventral tibial macroseta; smooth, curved, subequal claws with smooth, setiform, telotarsal process; not more than one pair of *ma* macrosetae on I–VII urotergites and up to one pair of *la* and *lp* on urotergites III–VII; with or without medial anterior or medial posterior macrosetae on urotergite VIII and abdominal segment IX; 3+3−2+2 *lp* macrosetae on urotergite VIII and 5+5−4+4 *lp* macrosetae on abdominal segment IX; first urosternite with 6+6 macrosetae (with 1+1 extra shorter, thinner macrosetae in *Libanocampacoiffaiti* Condé, 1955), 4+4 on urosternites II–VII and 1+1 on urosternite VIII; male first urosternite with area of continuous glandular *g_1_*-setae (split in two or absent in some species), subtrapezoidal appendages with glandular *a_1_* and *a_2_* setae (only *a_1_* in a few species); females without *g_1_* glandular setae (exception: C. (C.) franzi) with subcylindrical appendages with *a_1_* glandular setae. Cercal articles covered with whorls of macrosetae and setae in most species.

#### Remarks.

The genus *Campodea* is divided into five subgenera: *Campodea* s. str. Silvestri, 1932, *Dicampa* Silvestri, 1932, *Indocampa* Silvestri, 1933, *Monocampa* Silvestri, 1932 and Paurocampa Silvestri, 1932. The monotypic subgenus Hypercampa Silvestri, 1933, with Campodea (Hypercampa) essigi Silvestri, 1933 from California (USA), is excluded from the genus *Campodea* due to its thick and barbed telotarsal process.

#### Distribution.

Although they are distributed all over the world, the majority of species have been described in the Holarctic realm (Condé 1956).

### Subgenus﻿ Campodea

Taxon classificationAnimaliaDipluraCampodeidae

s. str. Silvestri, 1932

28718443-B69D-5EE5-A922-E90AC38BC236

#### Diagnosis.

Notal formula with 1+1 *ma*, 1+1 *la*, 1+1 *lp* macrosetae on pronotum and mesonotum, 1+1 *ma*, 1+1 *lp* on metanotum, besides a few species with 1+1 *ma*, 1+1 *lp* or 1+1 *la*, 1+1 *lp* on mesonotum and/or 0+0−1+1 *lp* macrosetae on metanotum; dorsal and lateral tarsal setae smooth; 0+0−1+1 *lp* macrosetae on urotergites IV–VII; from 0+0−1+1 *la* macrosetae on urotergites IV–VII; 0+0−1+1 *ma* or *mp* macrosetae on urotergites I–VIII; 3+3 *lp* macrosetae on urotergite VIII and 5+5 *lp* macrosetae on abdominal segment IX ; first urosternite of the male with an area of continuous (in a few species may be split in two patches) glandular *g_1_*-setae (absent in a few species and in seasonal periods), subtrapezoidal appendages with glandular *a_1_* and *a_2_* setae (*a_2_* glandular setae absent in a few species); females without *g_1_* glandular setae (with the exception of C. (C.) franzi) with subcylindrical appendages with *a_1_* glandular setae.

#### Remarks.

Campodea s. str. is the most diverse subgenus of Campodea so far, with 110 species.

#### Distribution.

Nearctic and Western Palearctic regions.

### ﻿1.- Campodea (Campodea) mondainii

Taxon classificationAnimaliaDipluraCampodeidae

Silvestri, 1931

6DBEBED6-EA6F-5F9A-879C-2C4C9E703EE9

#### Description.

Body 2.0 mm length; epicuticle smooth under optical microscope; antennae 0.4× as long as body with antennomeres 20–22 as long as wide; sensillum of the third antennomere unknown; pronotum with 3+3 (*ma*, *la*, *lp*), mesonotum with 3+3 (*ma*, *la*, *lp*), and metanotum with 2+2 (*ma*, *lp*) long barbed macrosetae; short clothing setae; marginal setae slightly longer than clothing setae and barbed; metathoracic leg 0.3× as long as body; urotergites I−VII with 1+1 *ma* short with bifurcated to several distal barbs macrosetae; urotergites IV−VII with 1+1 *la* short barbed macrosetae and 1+1 *lp* long barbed macrosetae; urosternite VIII with 3+3 *lp* long barbed macrosetae; urosternite I with 5+5 short macrosetae with few distal barbs; urosternite II−VII with 4+4 short macrosetae with few distal barbs; appendages of first urosternite short and subcylindrical in males and females but larger in males with more numerous glandular setae; cerci 0.45× as long as body with apparently eight articles plus basal one, covered with long macrosetae.

#### Habitat.

Deep layers in soil, probably an endogean species.

#### Distribution.

China (Hubei, Jiangsu, Zhejiang, Anhui, Hunan, Guizhou, Yunnan, Guangxi, Beijing, Henan, and Shandong) and Korea.

#### References.

Silvestri (1931).

### ﻿2.- Campodea (Campodea) ishii

Taxon classificationAnimaliaDipluraCampodeidae

Silvestri, 1931

244C89BB-20CC-59BF-9E25-01C5545C587F

#### Description.

Body 1.6 (juvenile) to 3.25 (adult) mm length; epicuticle smooth under optical microscope; antennae 0.5× as long as body with 20 (19 in juvenile) antennomeres as long as wide; sensillum of the third antennomere unknown; pronotum, mesonotum and metanotum with 3+3 (*ma*, *la*, *lp*), 3+3 (*ma*, *la*, *lp*), 2+2 (*ma*, *lp*) relatively long barbed macrosetae; short clothing setae; marginal setae slightly longer than clothing setae; metathoracic leg 0.3× as long as body; urotergites II−VII with 1+1 *ma* barbed macrosetae from short to much longer towards the posterior urotergites; urotergites IV−VII 1+1 *lp* long barbed macrosetae; urotergites V−VII 1+1 *la* short barbed macrosetae; urotergite VIII with 1+1 *mp* and 3+3 *lp* barbed macrosetae. urosternite I with 6+6 (5+5 in juveniles) short macrosetae with few distal barbs; urosternites II−VII with 4+4 short macrosetae with few distal barbs; appendages of first urosternite short and subcylindrical in females; males unknown; cerci 0.7× (juvenile)−0.8× (adult) as long as body with apparently ten articles plus basal one, all articles covered with numerous short clothing setae, and long macrosetae on basal articles.

#### Habitat.

Deep layers in soil, probably an endogean species.

#### Distribution.

East Asia: China (Shanghai, Anhui, and Guizhou), Korea and Japan.

#### References.

Silvestri (1931).

### ﻿3.- Campodea (?) pagei

Taxon classificationAnimaliaDipluraCampodeidae

 Silvestri, 1931

CC53F7B1-5123-5261-89AB-E8D962984453

#### Description.

Body 3.5 mm length; epicuticle no describe; antennae 0.4× shorter than the body length with 19 or 20 antennomeres as long as wide; sensillum of the third antennomere unknown;-pronotum with 2+2 (*ma*, *lp_3_*) short thick macrosetae with tiny distal barbs; short and thick clothing setae with tiny apical barb; marginal setae slightly longer than clothing setae with tiny distal barbs; metathoracic leg 0.3× as long as body; non macrosetae on urotergites I−VIII plus abdominal IX−X segments except a few strong setae probably macrosetae on posterior margin of abdominal segment X; urosternite I with 5+5 short macrosetae bearing few distal barbs; urosternites II−VII with 3+3 short macrosetae with few distal barbs; appendages of first urosternite subcylindrical slightly enlarged in males; cerci 0.45× as long as body with apparently ≤ 25 primary article plus basal one, covered with barbed setae much longer on distal articles.

#### Habitat.

Deep layers in soil, probably an endogean species.

#### Distribution.

Endemic in China (Hong Kong and Guangdong).

#### References.

Silvestri (1931).

#### Note.

At present we considered this species as belonging to the genus *Campodea*, but unknown subgenus. *Campodeapagei* lacks macrosetae on the abdominal segments, except for the tenth abdominal segment that supports a row of posterior macrosetae. This feature is completely unknown in other *Campodea* species. Nevertheless, the study of newly collected material is necessary to make further taxonomic decisions.

### ﻿Genus Metriocampa

Taxon classificationAnimaliaDipluraCampodeidae

Silvestri, 1912

497FC731-1D3D-5331-873C-34BB12303DE8

#### Diagnosis.

*Tricampa* Silvestri, 1912 is included in the genus *Metriocampa*, rejecting the artificial classification proposed by Paclt for both taxa (Condé and Geeraert 1962). The taxonomic difference between *Metriocampa* without *la* pronotal macrosetae and *Tricampa* with *la* pronotal macroseta seems not a valid reason for splitting both genera due to the similarities in other features: at most pronotum with 3+3 (*ma*, *la*, *lp_3_*), mesonotum 2+2 (*ma*, *la*), and metanotum 1+1 (*ma*) macrosetae; subequal simple, slightly curved claws, with or without a tiny latero-ventral spine; absence of lateral processes on pretarsus or with short setiform ones in *Metriocampavandykei* Silvestri, 1933 and *Metriocampaparadoxa* Condé & Geeraert, 1962; 1+1−2+2 *lp* macrosetae on urotergite VIII, 3+3−4+4 *lp* macrosetae on abdominal segment IX, and similarities in abdominal macrosetae patterns.

#### Remarks.

A total of 19 species have already been described.

#### Distribution.

Eastern Palearctic and Nearctic regions (Condé 1956; Sendra et al. 2021a)

### ﻿4.- Metriocampa kuwayamai

Taxon classificationAnimaliaDipluraCampodeidae

Silvestri, 1931

403F23B7-3434-50B6-8A82-32CBB6F8279C

#### Description.

Body 3 mm length; epicuticle smooth under optical microscope; antennae 0.3× as long as body with 19−22 antennomeres as long as wide; sensillum of the third antennomere unknown; pronotum with 2+2 (*ma*, *lp_3_*) macrosetae, mesonotum 2+2 (*ma*, *la*) and metanotum with 1+1 (*ma*) relatively short barbed macrosetae, except the longest *lp_3_*; short clothing setae; marginal setae longer than clothing setae; metathoracic leg 0.3× as long as body; urotergite VIII at least 1+1 *lp* barbed macrosetae; urosternite I with 5+5 short macrosetae with few distal barbs; urosternites II-VII with 4+4 short macrosetae with few distal barbs; appendages of first urosternite short and subcylindrical but much larger in males; first urosternite in males bearing a field of *g_1_* glandular setae on posterior position; cerci 0.7× as long as body with > 15 articles plus basal one, covered with long barbed macrosetae and clothing setae.

#### Habitat.

Deep layers in soil, probably an endogean species.

#### Distribution.

Eastern Asia, in China (Zhejiang, Anhui, Hunan, Jilin, Liaoning, Beijing, Henan, and Shanxi) and Japan.

#### References.

Silvestri (1931).

### ﻿5.- Metriocampa matsumurae

Taxon classificationAnimaliaDipluraCampodeidae

Silvestri, 1931

061A687A-172F-56BE-9316-4AA7A1C225D8

#### Description.

Body 3.5 mm length; epicuticle smooth under optical microscope; short and thin clothing setae; antennae 0.4× as long as body with 19−22 antennomeres as long as wide; not able to observe the third antennomere sensillum due to the poor condition of the specimen observed; pronotum with 2+2 (*ma*, *lp_3_*), and mesonotum with 1+1 (*ma*) relatively short macrosetae, except the longest *lp_3_*; short clothing setae; marginal setae longer than clothing setae; metathoracic leg 0.4× as long as body; urotergite VIII with 2+2 *lp* macrosetae; abdominal segment IX with 4+4 *lp* macrosetae; all urotergal macrosetae long and barbed; urosternite I with 5+5 short macrosetae with few distal barbs; urosternite II-VII with 4+4 short macrosetae bearing few distal barbs; appendages of first urosternite short and subcylindrical in females and much larger in males; first urosternite in males bearing a field of *g_1_* glandular setae on posterior position; cerci as longer as the body length with 12 primary articles plus basal one, covered with long barbed macrosetae and clothing setae.

#### Habitat.

Deep layers in soil, probably an endogean species.

#### Distribution.

Eastern Asia: China (Beijing and Shandong), Korea, and Japan.

#### References.

Silvestri (1931).

### ﻿6.- Metriocampa packardi

Taxon classificationAnimaliaDipluraCampodeidae

Silvestri, 1912

FDD4B288-EDB7-5719-A2C2-48D39AF905D3

#### Description.

Body 2.2−4.3 mm (adults) and 1.5−2.0 mm (juveniles) length; epicuticle smooth under optical microscope; antennae 0.4× as long as body with 18−25 antennomeres as long as wide; bacilliform sensillum on sternal third antennomere; pronotum with 2+2 (*ma*, *lp_3_*) barbed macrosetae; short clothing setae; marginal setae longer than clothing setae; metathoracic leg 0.3× as long as body; no dorsal femoral macrosetae nor sternal tibial macrosetae; urotergite VIII 2+2 *lp* barbed macrosetae, abdominal segment IX with 4+4 *lp* barbed macrosetae; urosternite I with 4+4−5+5 short macrosetae with few distal barbs; urosternite II−VII with 4+4 short macrosetae with few distal barbs; appendages of first urosternite short and subcylindrical in females and males enlarged up globous in males with *a_1_* and *a_2_* glandular setae; first urosternite in males bearing a field of *g_1_* glandular setae on posterior position; cerci 0.8× as long as body length with up to 20 articles plus basal one, covered with long barbed macrosetae and clothing setae.

#### Habitat.

Upper layers of the soil, among leaves in forest soils.

#### Distribution.

Holarctic realm, including China (Jilin) and North America.

#### References.

Silvestri (1911, 1933a); Condé and Thomas (1957); Bareth and Condé (1958).

### ﻿7.- Metriocampa sahi

Taxon classificationAnimaliaDipluraCampodeidae

Silvestri, 1931

417EFEB9-85F2-51F7-A5E0-DC5643EB19BF

#### Description.

Body 2.2−3.5 mm length; epicuticle with apparently no microdenticles in optical microscope; thick and short clothing setae; antennae 0.5× as long as body with 21−23 antennomeres as long as wide; tiny coniform ventral sensillum on third antennomere; pronotum with 2+2 (*ma*, *lp_3_*) and mesonotum with 1+1 (*ma*) relatively long robust macrosetae with a few distal barbs; short thick clothing setae with a distal barb; marginal setae slightly longer and more robust than clothing setae; metathoracic leg 0.3× as long as body; urotergite VIII with 2+2 *lp* barbed macrosetae; urosternite I with 5+5 short macrosetae with few distal barbs; urosternite II−VII with 4+4 short macrosetae with few distal barbs; appendages of first urosternite short and subcylindrical in females enlarged in males; males appendages with *a_1_* and *a_2_* glandular setae and a posterior field of *g_1_* glandular setae on posterior position on first urosternite; cerci 0.8× as long as body with ~ 17 articles plus basal one, covered with long thin macrosetae with distal barbs.

#### Habitat.

Deep layers in soil, probably an endogean species.

#### Distribution.

Endemic in China (Fujian, Shanghai, Hunan, and Sichuan).

#### References.

Silvestri (1931).

### ﻿8.- Metriocampa urumqiensis

Taxon classificationAnimaliaDipluraCampodeidae

Chou & Chen, 1980

2C83C66A-925F-5240-9058-451B5EEE1232

**Figure 1.**
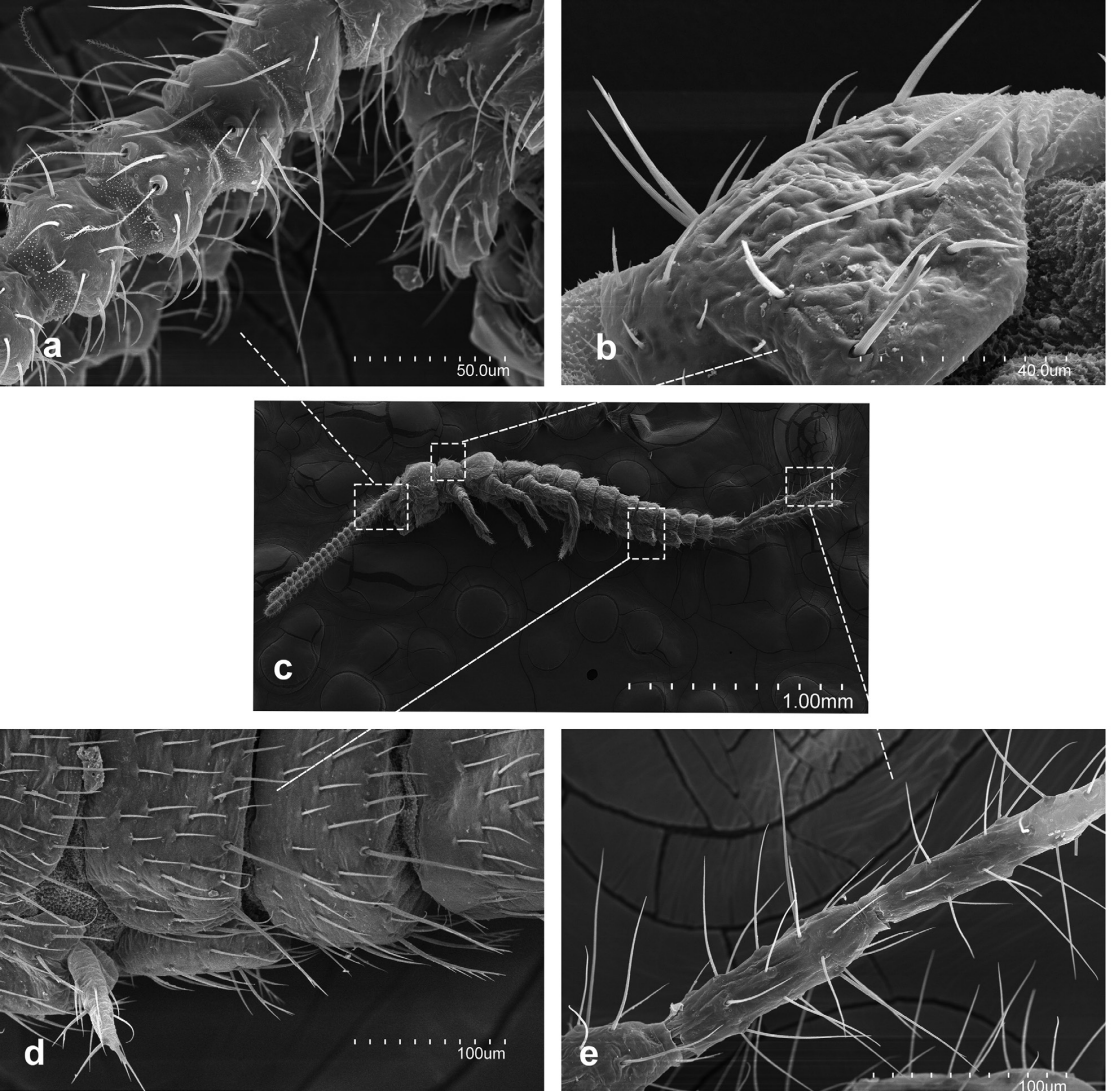
*Metriocampaurumqiensis* Chou & Chen, 1980 **a** proximal part of antennae **b** pronotum, lateral view **c** habitus **d** abdominal segment seventh to tenth, lateral view **e** medial part of a cercus.

**Figure 2.**
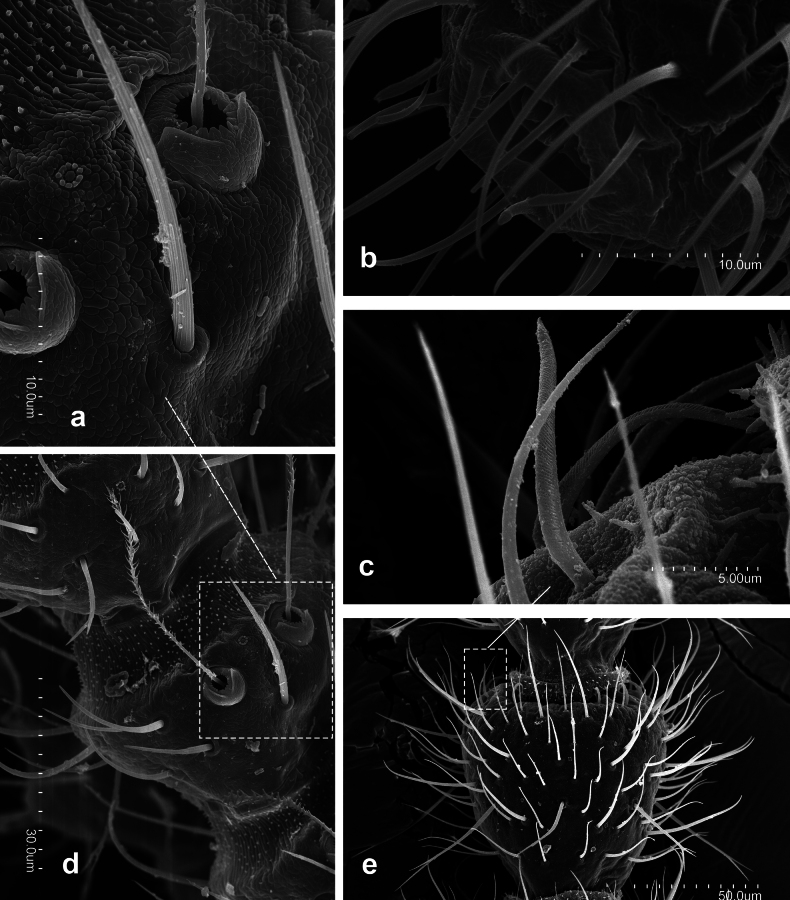
*Metriocampaurumqiensis* Chou & Chen, 1980 **a** dorsal portion of the third antennomere, with its trichobothria **b** distal part of apical antennomere with its cupuliform organ **c** latero-distal part of a medial antennomere, with some gouge sensilla **d** third antennomeres **e** medial antennomere.

**Figure 3.**
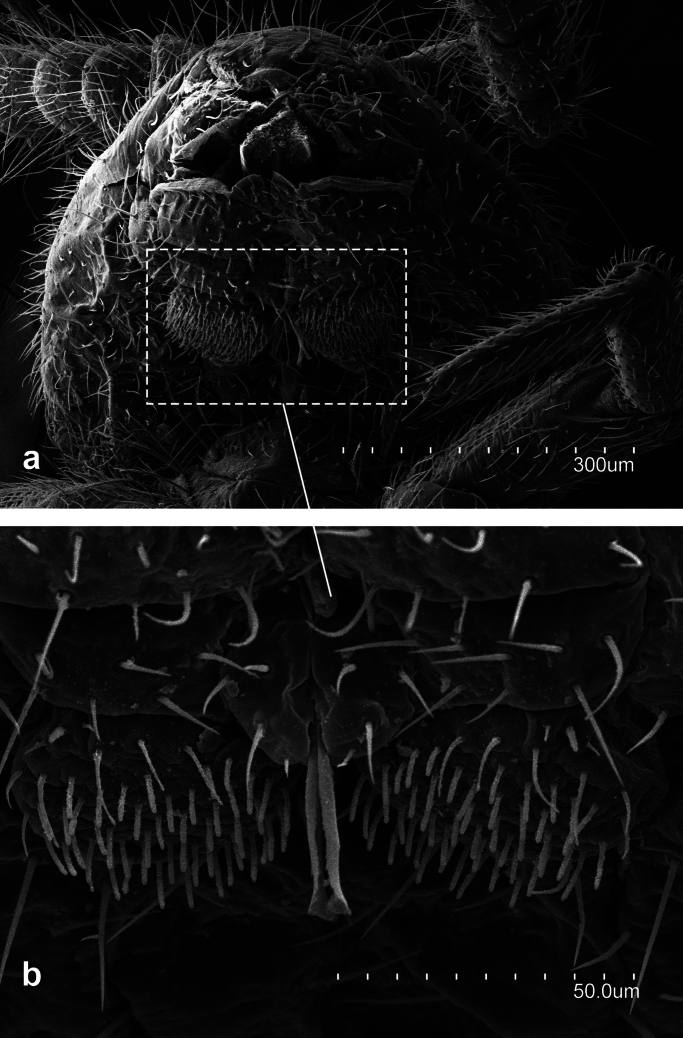
*Metriocampaurumqiensis* Chou & Chen, 1980 **a** head, ventral view **b** anterior portion of the labium with their labial palps (*sétigère plaque*) and palpiform processes.

**Figure 4.**
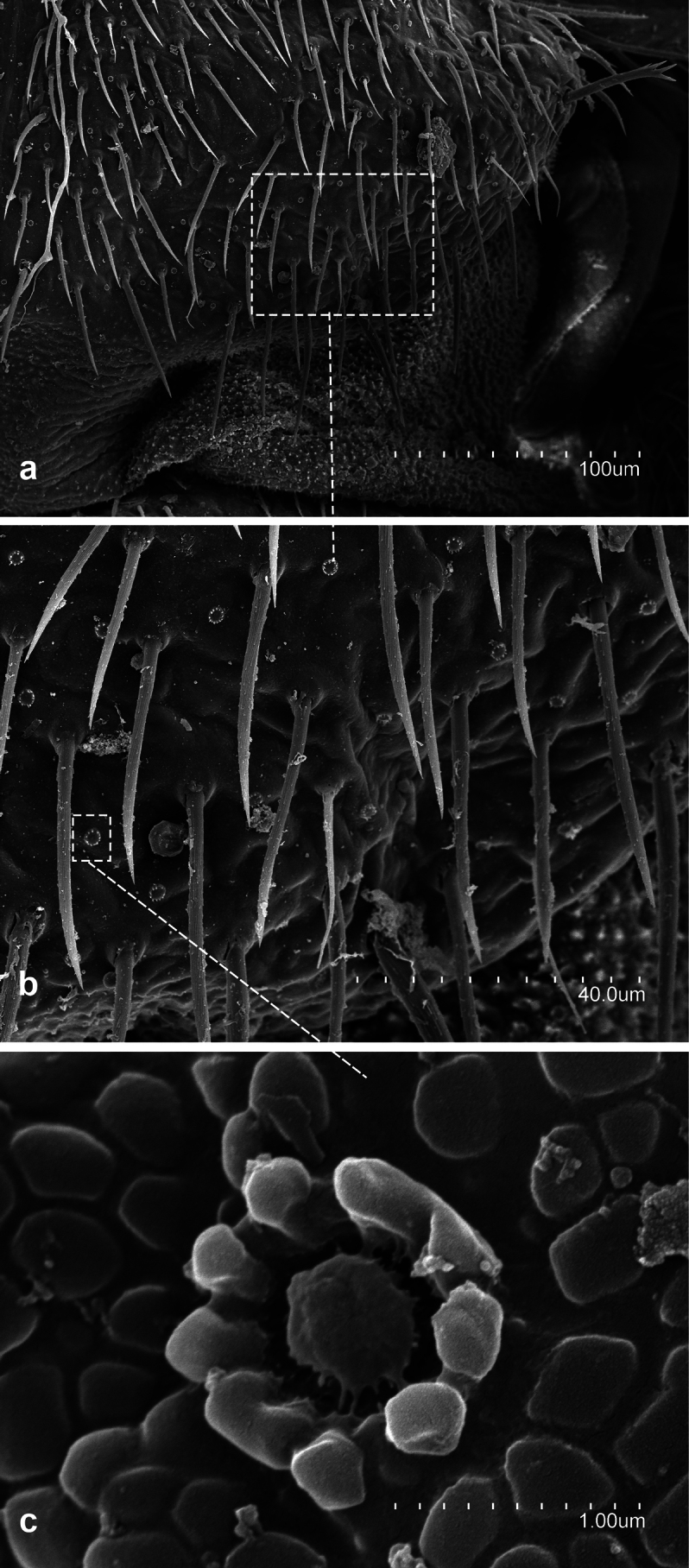
*Metriocampaurumqiensis* Chou & Chen, 1980 **a** lateral part of the pronotum **b** latero-posterior view of the pronotum **c** detail of rosette-like formation.

**Figure 5.**
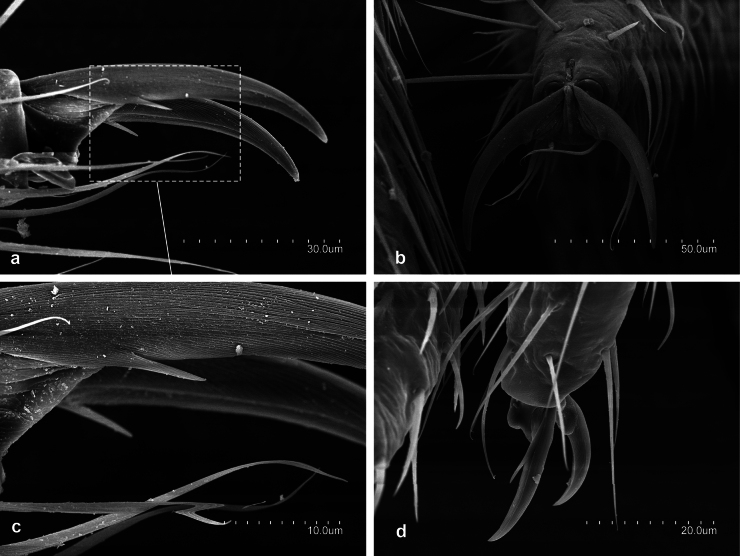
*Metriocampaurumqiensis* Chou & Chen, 1980 **a** lateral view of pretarsus **b** frontal view of pretarsus **c** detail of pretarsus, medial portion of the claws **d** pretarsus, latero-tergal view.

**Figure 6.**
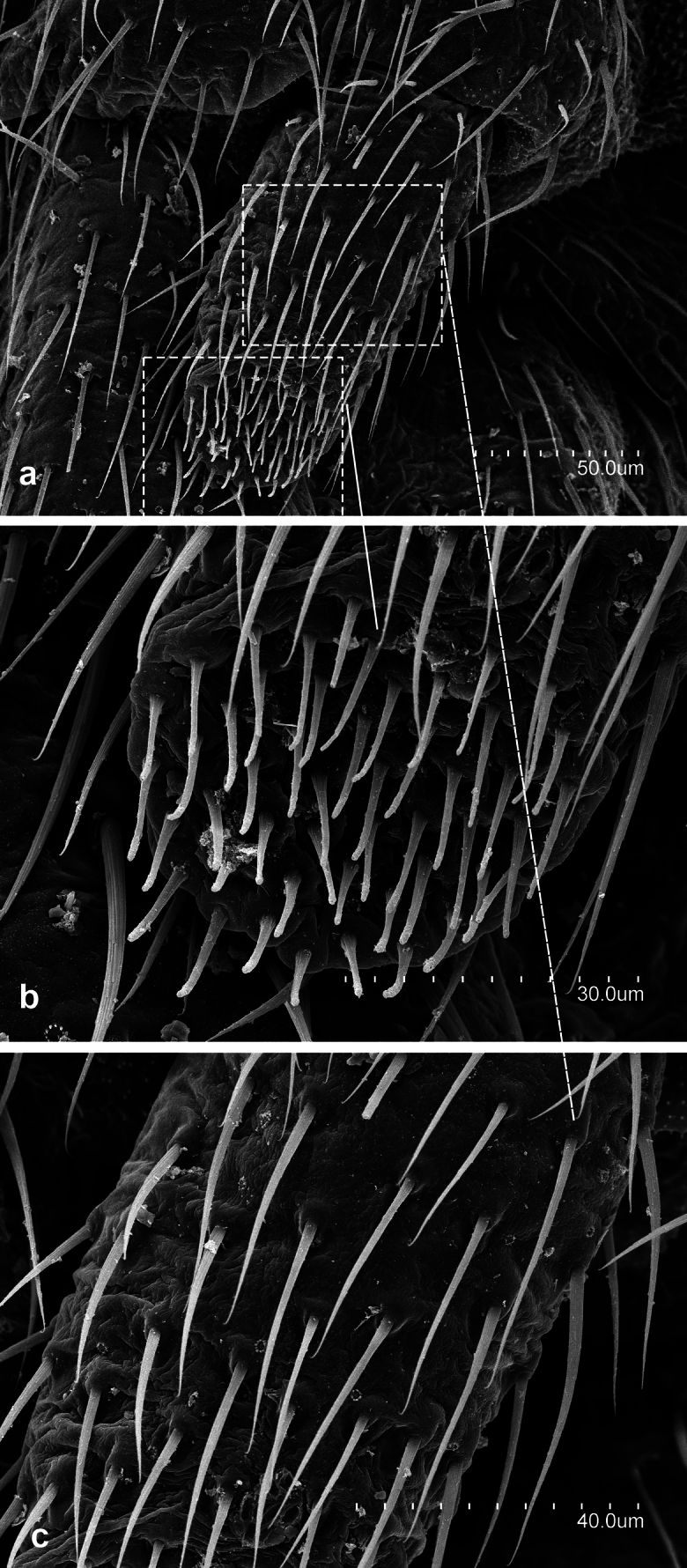
*Metriocampaurumqiensis* Chou & Chen, 1980 male specimen **a** latero-posterior portion of the first urosternite **b** distal part of the appendage of the first urosternite **c** medial portion of the appendage of the first urosternite.

**Figure 7.**
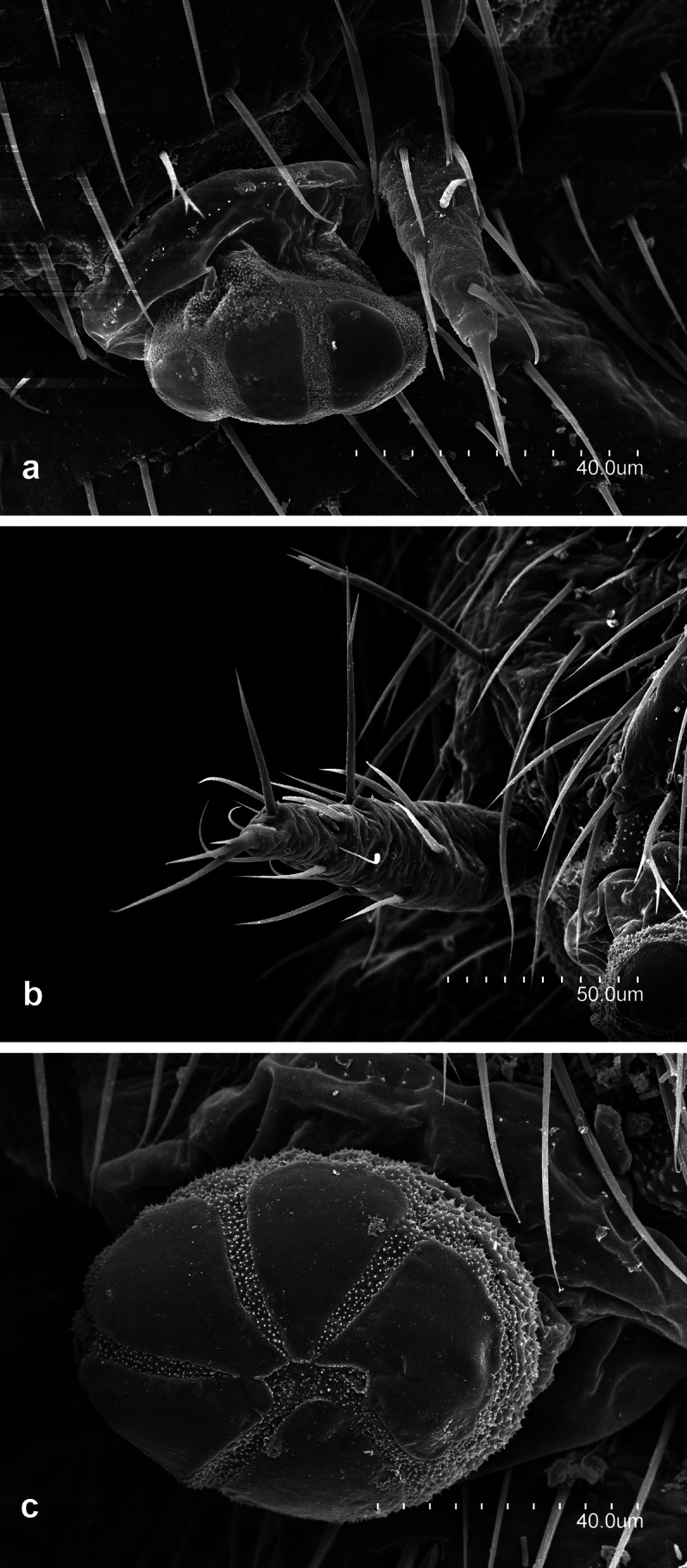
*Metriocampaurumqiensis* Chou & Chen,1980 **a** latero-posterior portion of medial urosternite **b** stylus of a medial urosternite **c** eversible vesicle of a medial urosternite.

Figs 1 , 2 , 3 , 4 , 5 , 6 , 7

#### Description.

Body 2.1−4.4 mm length; epicuticle without microdenticles using optical microscope describe; antennae 0.3−0.6× as long as body with 16−27 antennomeres as long as wide; ventral sensillum small and subcylindrical on third antennomere; pronotum, mesonotum, and metanotum with 3+3 (*ma*, *la*, *lp_3_*), 2+2 (*ma*, *la*), 1+1 (*ma*) relatively short bifurcated macrosetae, except the longest *lp_3_*; short clothing setae; marginal setae longer than clothing setae; metathoracic leg 0.3× as long as body; urotergite VIII with 2+2 *lp* macrosetae; abdominal segment IX with 4+4 *lp* macrosetae; urosternite I with 5+5 short bifurcated macrosetae; urosternite II−VII with 4+4 short bifurcated macrosetae; appendages of first urosternite short and subcylindrical in females and males, but in males larger appendages bearing *a_1_* and *a_2_* glandular setae and male first urosternite has a field with three or four rows of *g_1_* glandular setae on posterior area; cerci 0.4× as long as body with apparently eight articles plus basal one, covered with long barbed and long smooth clothing setae.

#### Habitat.

Upper layers of the soil, among leaves in forest soils.

#### Distribution.

Endemic in China (Xinjiang, Qinghai, Gansu, Ningxia, Sichuan, and Shaanxi).

#### References.

Chou and Chen (1980).

### ﻿9.- Metriocampa wuyanlinensis

Taxon classificationAnimaliaDipluraCampodeidae

Xie & Yang, 1991

200FD6A7-4E99-5DD0-BF82-5B73B6E54791

#### Description.

Body 2.0−5.0 mm length; epicuticle with microdenticles at optical microscope; antennae 0.6× as long as body with 22−25 antennomeres as long as wide; ventral small subcylindrical third antennomere sensillum; pronotum, mesonotum, and metanotum with 2+2 (*ma*, *lp_3_*), 2+2 (*ma*, *la*), 1+1 (*ma*) relatively short macrosetae with a distal barb, except the longest *lp_3_*; short clothing setae; marginal setae longer than clothing setae; metathoracic leg 0.3× as long as body; urotergites VIII 2+2 *lp*; abdominal segment IX with 4+4 *lp* macrosetae; urosternite I with 5+5 short macrosetae with few distal barbs; urosternite II−VII with 4+4 short macrosetae with few distal barbs; appendages of first urosternite short and subcylindrical in females and males; males with a narrow field of *g_1_* glandular setae on posterior area of first urosternite.

#### Habitat.

Soil.

#### Distribution.

Endemic in China (Zhejiang and Sichuan).

#### References.

Xie and Yang (1991).

### ﻿Genus Leniwytsmania

Taxon classificationAnimaliaDipluraCampodeidae

Paclt, 1957

D9D9F9CC-4458-5BBA-ACEB-B7EB484C94E7

#### Diagnosis.

*Leniwytsmania* was proposed by Paclt (1957) based on the setiform and setiform lateral processes with barbs in two species considered in the paraphyletic *Eutrichocampa*. Another feature of *Leniwytsmania* is the reduction of notal macrosetae, as happened in the subgenera of *Campodea*: pronotum, mesonotum, and metanotum with 3+3 (*ma*, *la*, *lp_3_*), 0 or 1+1 (*la*) and no macrosetae.

#### Remarks.

Two species were included by Paclt (1957) into *Leniwytsmania*.

#### Distribution.

Two distant areas of distribution, *Leniwytsmaniahelvetica* (Wygodzinsky, 1941) in central Europe and *Leniwytsmaniaorientalis* (Silvestri, 1931) in China.

### ﻿10.- Leniwytsmania orientalis

Taxon classificationAnimaliaDipluraCampodeidae

(Silvestri, 1931)

0DC9D9CD-A0E1-5430-AD39-169FE7B759AC

Figs 8 , 9 , 10

#### Description.

Body 1.9−2.4 mm length; epicuticle with microdenticles at optical microscope; antennae 0.5–0.6× as long as body with 18−21 antennomeres as long as wide; small subcylindrical tergal sensillum on third antennomere; pronotum and mesonotum with 3+3 (*ma*, *la*, *lp_3_*) and 1+1 (*la*) relatively short barbed macrosetae, *lp_3_* the longest; short clothing setae; marginal setae slightly more robust and longer than clothing setae with one or three barbs; metathoracic leg 0.4× as long as body; claws with small lateral crests and ventral body claws with tiny grooves; short lateral processes with terminal barbs; urotergites V−VII with 1+1 *lp* barbed macrosetae; urotergite VIII with 3+3 *lp*, urotergites; abdominal segment IX with 5+5 *lp*, all macrosetae long barbed macrosetae; urosternite I with 6+6 short bifurcated macrosetae; urosternite II−VII with 4+4 short bifurcated macrosetae;-appendages of first urosternite short and subcylindrical in females, subtrapezoidal in males; first urosternite in males bearing a field of *g_1_* glandular setae on posterior portion; cerci 0.8× as long as body with nine articles plus basal one, covered with long macrosetae, well barbed on external side macrosetae and poorly barbed on internal side macrosetae, plus a few clothing setae.

**Figure 8.**
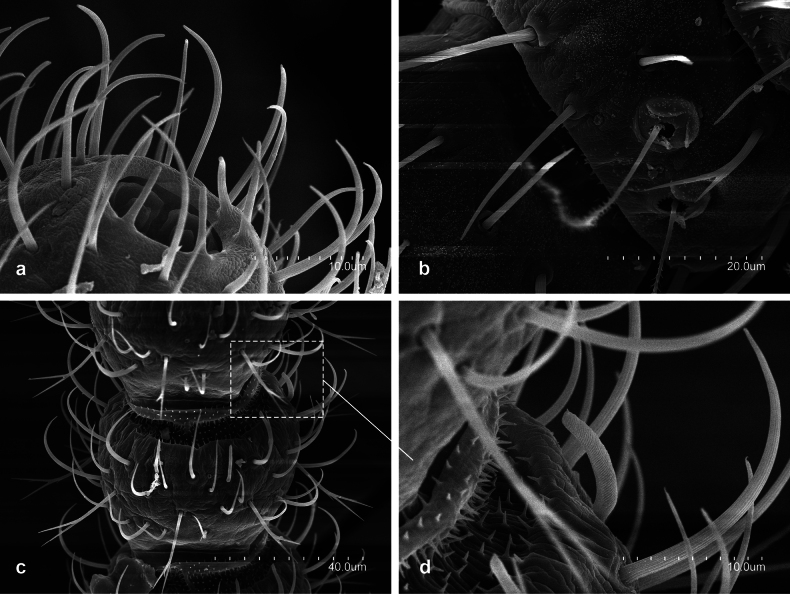
*Leniwytsmaniaorientalis* Silvestri, 1931 **a** apical antennomere with its cupuliform organ **b** fourth antennomere with their trichobothria **c** medial antennomeres **d** latero-distal portion of medial antennomere with a gouge sensilla.

**Figure 9.**
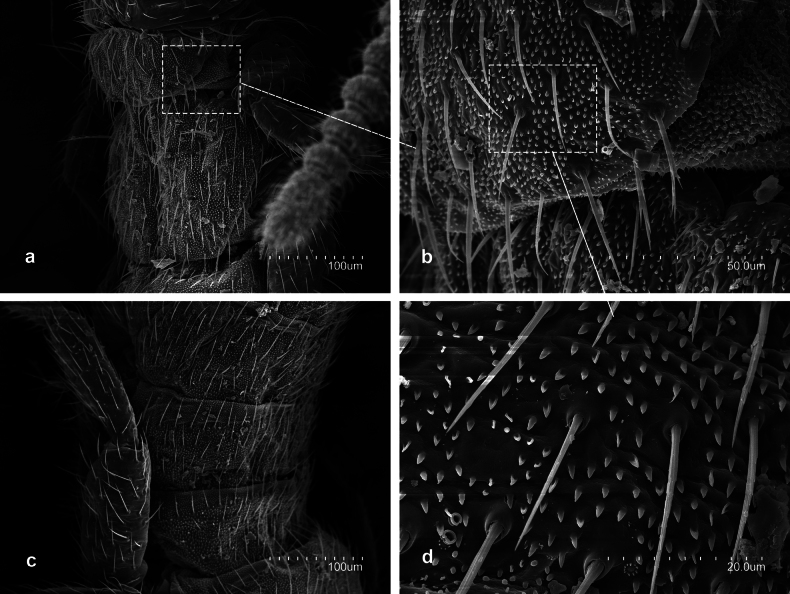
*Leniwytsmaniaorientalis* Silvestri, 1931 **a** Prothorax and Metathorax **b** latero-posterior portion of metathorax **c** first to third abdominal segments and part of one metathoracic leg **d** detail of pronotum.

**Figure 10.**
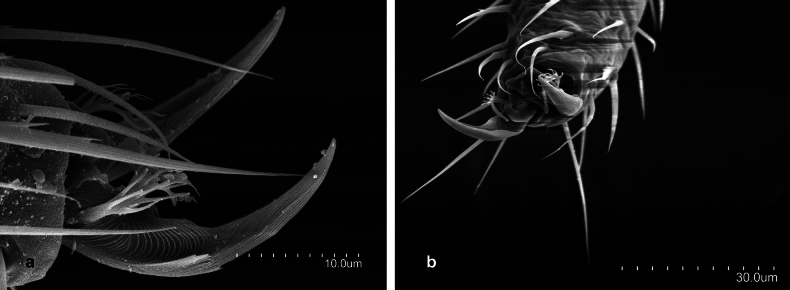
*Leniwytsmaniaorientalis* Silvestri, 1931 **a** Pretarsus, lateral view **b** Pretarsus, latero-ventral view.

#### Habitat.

Deep layers in soil, probably an endogean species.

#### Distribution.

Endemic in China (Yunnan, Hunan, Hubei, Sichuan, Guizhou, and Guangxi).

#### References.

Silvestri (1931); Paclt (1957).

### ﻿Genus Pseudolibanocampa

Taxon classificationAnimaliaDipluraCampodeidae

Xie & Yang, 1991

57C068A2-313C-56F5-90B0-C3EC20937A79

#### Diagnosis.

Pronotum and mesonotum with 2+2 (*ma*, *lp_3_*), 1+1 (*la*); pretarsus with elbow-like claws; laminar lateral process ending with barbs; urotergites V-VII 1+1 *lp*; urotergites VIII with 2+2 *lp*; abdominal segment IX with 4+4 *lp* macrosetae.

#### Remarks.

A monotype genus.

#### Distribution.

Endemic in China (Xie and Yang 1991).

### ﻿11.- Pseudolibanocampa sinensis

Taxon classificationAnimaliaDipluraCampodeidae

Xie & Yang, 1991

5D80CC6E-745C-5C18-A149-A413840562B4

#### Description.

Body 1,9 mm length; epicuticle with microdenticles under optical microscope; antennae of body with 15−19 antennomeres as long as wide; bacilliform sternal sensillum on the third antennomere; pronotum and mesonotum with 2+2 (*ma*, *lp_3_*), 1+1 (*la*) relatively short macrosetae; short clothing setae with a distal barb; marginal setae similar to clothing setae; metathoracic leg 0.2× shorter of the body length; calcars short with one distal barb; pretarsus with elbow-like claws; laminar lateral process ending with barbs; urotergites V-VII 1+1 *lp*; urotergites VIII with 2+2 *lp*; abdominal segment IX with 4+4 *lp* macrosetae, relatively short macrosetae with a few distal macrosetae.

#### Habitat.

Soil.

#### Distribution.

Endemic in China (Yunnan, Guangxi, and Guangdong).

#### References.

Xie and Yang (1991).

### ﻿Genus Pacificampa

Taxon classificationAnimaliaDipluraCampodeidae

Chevrizov, 1978

3E304557-E641-54CB-90B2-0C93A43C84B0

#### Diagnosis.

Thoracic macrosetae with no more than 3+3 macrosetae on pronotum, 4+4 on mesonotum (2+2 *lp* included), and 2+2 on metanotum (1+1 *lp* included). One dorsal macroseta on metathoracic femora and one or two on tibia. Subequal elbowed claws with ridges on dorsal side that look like very small lateral crests under the optical microscope; without lateral processes. Urotergites V–VII with no more than 1+1 *mp*, 1+1 *la*, and 2+2 *lp* macrosetae and on urotergite VIII with no more than 1+1 *mp* and 3+3 *lp* macrosetae. Up to 7+7 macrosetae on first urosternite, 5+5 macrosetae on urosternites 2–7, and 1+1 macrosetae on eighth urosternite. First urosternite in males with thick appendages bearing large field of glandular *a_1_* setae.

#### Remarks.

Three species belong to this genus (Chevrizov 1978; Sendra et al. 2021b).

#### Distribution.

Eastern of Asia (Chevrizov 1978; Sendra et al. 2021b).

### ﻿12.- Pacificampa wudonghuii

Taxon classificationAnimaliaDipluraCampodeidae

Sendra, 2021

61DB6C75-A67A-5709-9408-640B5B0CB905

#### Description.

Body length 6.5–6.9 (adults) mm, 3.9 (juvenile) mm. cuticle smooth under optical microscope but clearly reticulated under high magnifications with scattered external glands; smooth clothing setae; bacilliform sensillum in ventral position of the third antennomere, between *c–d* macrosetae; plain frontal process. Pronotum has 1+1 *ma*, 1+1 *la*, 1+1 *lp* macrosetae; mesonotum has 1+1 *ma*, 1+1 *la*, and 2+2 *lp* macrosetae; and metanotum has 1+1 *ma* and 1+1 *lp* macrosetae; long barbed macrosetae; barbed marginal setae longer than clothing setae; elongated legs reach posterior border of seventh abdominal segment; mesothoracic and metathoracic femora have one dorsal macroseta each; calcars with two or three long barbs; prothoracic and mesothoracic tibia with one short ventral macrosetae with one apical barb and two in metathoracic tibia; subequal elbowed claws with smooth ventral surface ridged on dorsal side that can be mistaken for lateral crests under optical microscopes, between a blunt unguiculus and without lateral processes; distribution of abdominal macrosetae on urotergites 0−1+1 *ma* on III; 1+1 *ma*, 1+1 *la*, and 2+2 *lp* on IV–VII; 1+1 *mp* and 3+3 *lp* on VIII; and 1+1 *mp* and 5+5 *lp* on abdominal segment IX; *ma* and *la* macrosetae with barbs and shorter than *mp* and *lp* barbed macrosetae; urosternite I with 7+7 macrosetae; urosternites II–VII with 4+4 macrosetae; urosternite VIII with 1+1 macrosetae; stylus setae smooth; female urosternite I with subcylindrical appendages with glandular *a_1_* setae; male with thick short, and subcylindrical appendages, each with large apical field of glandular *a_1_* setae.

#### Habitat.

Deep subterranean ecosystems, in caves habitats.

#### Distribution.

Endemic in China (Liaoning).

#### References.

Sendra et al. (2021b).

### ﻿Subfamily Plusiocampinae

Taxon classificationAnimaliaDipluraCampodeidae

Paclt, 1957

79668158-B959-5666-BEA4-005E374F6621

#### Diagnosis.

The pronotum never has fewer than 4+4 macrosetae, usually not less than 1+1 *ma*, 1+1 *la*, and 2+2 *lp*, except a few species with a distinctive macrosetae pattern (1+1 *ma*, 2+2 or 3+3 *la*, and 1+1 *lp*, as in *Paratachycampa* Wygodzinsky, 1944 and two species of Plusiocampa (Stygiocampa) Silvestri, 1934, P. (S.) christiani Condé & Bareth, 1996 and P. (S.) denisi Condé, 1947

#### Remarks.

At present, 103 species have been described belonging to 15 genera (Condé 1956; Sendra et al. 2021a).

#### Distribution.

Mostly distributed in Palearctic region, this genus is also recognized in the Nearctic, with a single genus in the Ethiopian realm (*Silvestricampa* Condé, 1950).

### ﻿Genus Plusiocampa

Taxon classificationAnimaliaDipluraCampodeidae

Silvestri, 1912

C145B15E-D7B9-5A53-8DC3-E759C0C42E29

#### Diagnosis.

Apparently smooth cuticle, usually reticulated at high magnification, rosette-like pores absent. Head with a frontal process with or without tuberculate setae. In non-troglomorphic species, cupuliform organ with four or five spheroidal olfactory chemoreceptors in polygonal net, with pore surface made by at least one cup-shaped fold and a central structure ending in a terminal pore. In troglobiomorphic species, spheroidal olfactory chemoreceptor with more folds in spiral, radial, or other complex shapes and a more visible polygonal net with pore surface. Sensillum of third antennomere in ventral position. Meso- and metathorax with a few macrosetae but frequently with medial anterior, lateral anterior, lateral posterior, and medial posterior (exceptionally medial intermediate and lateral intermediate) macrosetae. Femur with one to five dorsal macrosetae; tibia with one to three ventral macrosetae. Elbow-like claws usually with large lateral crests and setiform smooth lateral processes, rarely with a few barbs on proximal part. Abdomen with lateral anterior and posterior macrosetae and never with medial anterior macrosetae. Sternal macrosetae: sternite I with 6+6 up to 60 (in total) macrosetae; sternites II–VII with 4+4 to up to 14+14 macrosetae; sternite VIII with 2+2 up to 4+4 macrosetae. Secondary sexual differences in shape of the first urosternite appendages and the number of glandular setae.

#### Remarks.

A genus with five subgenera and a total of 71 species described (Condé 1956; Sendra et al. 2021a).

#### Distribution.

This genus is primarily distributed in the Western Palearctic, with the exception of *Plusiocampasinensis*, which will require further review in the future.

### ﻿13.- Plusiocampa

Taxon classificationAnimaliaDipluraCampodeidae

(incer. sed.) sinensis Silvestri, 1931

C419F99A-DB61-5C8F-938B-893F724951FF

#### Description.

Body 1.6−2.3 mm length; cuticle smooth under optical microscope; antennae 0.5−0.7× as long as body with 20−26 antennomeres as long as wide; large bacilliform tergal sensillum of the third antennomere; four plain sensilla in cupuliform organ; pronotum with 4+4 (*ma*, *la*, *lp_1_*_,*3*_) macrosetae, mesonotum with 4+4 (*ma*, *la*, *lp_2_*_,*3*_) macrosetae and metanotum 3+3 (*ma*, *lp_2_*_,*3*_) with (all notal macrosetae long and barbed); with few long clothing setae; marginal setae slightly longer than clothing setae and wit a few distal barbs; metathoracic leg 0.3−0.4× as long as body; two dorsal femoral macrosetae; one short sternal tibial macrosetae; smooth setiform lateral processes urotergites I−III with 1+1 *post* macrosetae; urotergite IV with 1+1−2+2 macrosetae, urotergites V−VII with 3+3−4+4 *post* macrosetae and urotergites VIII with 5+5 *post* macrosetae and abdominal segment IX with 7+7 *post* long barbed macrosetae; urosternite I with 6+6−9+9 macrosetae; urosternite II−VII with 4+4, urosternite VIII with 1+1 macrosetae; stylus of the *Campodea* pattern; appendages of urosternite I subcylindrical females, and suboval in males; appendages of males with and *a_2_* glandular setae; cerci 0.6−0.8× as long as body.

#### Habitat.

Deep layers in soil, probably an endogean species.

#### Distribution.

Endemic in China (Fujian, Guangdong, and Hong Kong).

#### References.

Condé (1993a), Silvestri (1931).

### ﻿Genus Cestocampa

Taxon classificationAnimaliaDipluraCampodeidae

Condé, 1956

387DEC7A-ECA2-5865-8EC0-48DAACC205EF

#### Diagnosis.

It is diagnosed exclusively by the presence of a laminar barbed process of the pretarsus, in addition to the macrosetae distribution pattern that matches that of the Plusiocampa (Plusiocampa).

#### Remarks.

Five species from soil and cave habitats (Sendra et al. 2012).

#### Distribution.

Four Mediterranean species and a single species in Eastern Asia (China), making it likely a paraphyletic genus (Sendra et al. 2012).

### ﻿14.- Cestocampa kashiensis

Taxon classificationAnimaliaDipluraCampodeidae

Chou & Chen, 1980

882700A3-D193-50E6-AA3B-C516842C07B7

#### Description.

Body 5.0 mm length; pronotum with 4+4 (*ma*, *la_3_*, *lp_1_*_,*3*_), mesonotum with 4+4 (*ma*, *la_3_*, *lp_2_*_,*3*_), and metanotum 3+3 (*ma*, *lp_2_*_,*3*_) long barbed macrosetae covered by short barbs; short clothing setae and longer thicker marginal setae; one sternal macrosetae on tibia; subequal plain claws with laminar and barbed lateral processes; anterior urotergites with 1+1 *post*, posterior urotergites with 4+4 *post* long well-barbed macrosetae; urosternite I with 6+6, II−VII with 4+4, and VIII with 1+1 barbed macrosetae; stylus with apical macroseta bifurcated and other poorly barbed setae.

#### Habitat.

Soil.

#### Distribution.

Endemic in China (Xinjiang).

#### References.

Chou and Chen (1980); Sendra et al. (2012).

### ﻿Genus Plutocampa

Taxon classificationAnimaliaDipluraCampodeidae

Chevrizov, 1978

B33A3C74-36CB-5057-B7CE-9838AB6B747A

#### Diagnosis.

Thick sensilla on third antennomere and labial palps. Thoracic macrosetae with at least 5+5 macrosetae on pronotum, 5+5 on mesonotum (1+1 *mp* included), and 4+4 on metanotum (1+1 *mp* included). Two dorsal macrosetae on the metathoracic femora and one on the tibia. Unequal elbowed claws with lateral crests without lateral processes. Urotergites V–VII with 4+4 *post* macrosetae and without *la* macrosetae. Up to 6+6 macrosetae on the first urosternite, 4+4 macrosetae on urosternites II–VII, and 1+1 macrosetae on the urosternite VIII (Chevrizov 1978; Sendra et al. 2021b).

#### Remarks.

A genus with three species with a tendency to occupy subterranean habitats (Sendra et al. 2021b).

#### Distribution.

Extreme East of Asia, in Russia and China (Chevrizov 1978; Sendra et al. 2021b).

### ﻿15.- Plutocampa methoria

Taxon classificationAnimaliaDipluraCampodeidae

Chevrizov, 1978

3D932F81-1D42-50D2-8B2B-D760F0FABCCF

#### Description.

Body length 5.4 mm; Smooth cuticle covered by thin smooth clothing setae; antennae appears regenerated and it has 23 antennomeres; thick sensillum in ventral position of the third antennomere; small pointed frontal process; labial palps have a large thick sensilla; pronotum with 1+1 *ma*, 2+2 *la*, 2+2 *lp_1_*_,*3*_, mesonotum with 1+1 *ma*, 2+2 (2+1) *la*, 2+2 *lp_2_*_,*3*_, 1+1 *mp*, and metanotum with 1+1 *ma*, 0–1+1 *la*, 2+2 *lp_2_*_,*3*_, 1+1 *mp* macrosetae; long thin notal macrosetae with fine barbs; marginal setae are as long and barbed as the macrosetae; metathoracic legs reach the posterior border of abdominal segments V or VI; femora has two long, barbed, dorsal macrosetae; tibia has one short ventral macroseta; pretarsus without lateral processes, with unequal elbowed claws and large lateral crests; urotergites I and II with 1+1 *post_1_*; urotergite III with 2+2 *post_1_*_,*2*_; urotergites IV–VII with 4+4 *post_1–4_*; urotergite VIII with 5+5 *post_1–5_* and abdominal segment IX with 7+7 *post*, macrosetae are long with barbs; urosternite I with 6+6, urosternites II–VII with 4+4, and urosternite VIII with 1+1 macrosetae; urosternal macrosetae shorter than urotergal macrosetae with long barbs; smooth stylar setae; appendages of the first urosternite (female) with large, short, and subcylindrical appendages, each with apical field of glandular *a_1_* setae.

#### Habitat.

In soil and also found in cave environments but without cave−adaptive features.

#### Distribution.

Eastern Asia, in the Far East of Russia and China (Liaoning).

#### References.

Chevrizov (1978); Sendra et al. (2021b).

### ﻿Genus Anisuracampa

Taxon classificationAnimaliaDipluraCampodeidae

Xie & Yang, 1991

74F537C4-47CF-54F1-B1A7-D5422FBDB83B

#### Diagnosis.

Mesonotum and metanotum with 1+1 medial anterior, none or 2+2 lateral anterior, and 2+2 to 4+4 lateral posterior macrosetae. Two or three dorsal macrosetae on metathoracic legs. Elbowed claws ventrally with long spiniform formations and apparently lateral crests. Laminar pretarsus lateral processes with long, broad barbs. Urotergites with 4+4–5+5 posterior macrosetae on urotergites V–VII. Eighth urosternite with 1+1 macrosetae. First urosternite with non–glandular setae bearing coniform or subtrapezoidal appendages (Sendra et al. 2021b; Xie and Yang 1991).

#### Remarks.

A genus with two species, one living in soil and the other in cave environments (Sendra et al. 2021b; Xie and Yang 1991).

#### Distribution.

East Asia, in China and Myanmar (Sendra et al. 2021b; Xie and Yang 1991).

### ﻿16.- Anisuracampa suoxiensis

Taxon classificationAnimaliaDipluraCampodeidae

Xie & Yang, 1991

B909D721-FA18-544D-9D7F-108802059811

#### Description.

Body 1.7−3.5 length; antenna 24 antennomeres; pronotum with 4+4 (*ma*, *la*, *lp_1_*_,*3*_), mesonotum with 5+5 (*ma*, *la_2_*_,*3*_, *lp_2_*_,*3*_), metanotum with 4+4 (*ma*, *la*, *lp_2_*_,*3*_) long barbed macrosetae; thin clothing setae; marginal setae similar to clothing setae; two dorsal macrosetae on femur of the third pair of legs; urotergites I−II with 1+1 *post*, III with 2+2 *post*, IV−VII with 4+4 *post* long barbed macrosetae.

#### Habitat.

Soil, probably edaphic species.

#### Distribution.

Endemic in China (Hunan).

#### References.

Xie and Yang (1991).

### ﻿Genus Whittencampa

Taxon classificationAnimaliaDipluraCampodeidae

Sendra & Deharveng, 2020

2B9505EB-B3A3-5A78-B6A3-4489225A1B88

#### Diagnosis.

Pronotum with 1+1 *ma*, 1+1 *la* and 2+2 *lp*, mesonotum and metanotum with 1+1 *ma*, 1+1 *la*, and 2+2 *lp* ; two dorsal femoral macrosetae; without tibial macrosetae; unequal claws with lateral-crests with extensions at the basal end of the posterior claw; two thick setiform pretarsal processes completely covered with long barbs with a tiny hook end; female urosternite I with coniform appendages thinner than male appendages, each bearing thin and long glandular *a_1_* setae in a distal field; male urosternite I with large and enlarged subcylindrical appendages, each bearing long glandular *a1* setae and a large field of thin and long glandular *a_2_* setae; male and female without glandular field on the posterior part of the first urosternite; 1+1 *post* urotergal macrosetae on urotergites III–IV, 4+4 *post* on V–VII, 5+5 *post* on VIII and 7+7 *post* on abdominal segment IX; 13+13–10+10 macrosetae on urosternite I, 5+5 on urosternites II–VII, and 1+1 macrosetae on urosternite VIII; large subtriangular ending of neuroglandular setae and epidermal glands on labial palps plus rosette-like glands similar to observed in the subfamily Campodeinae; eversible vesicles with a double sac.

#### Remarks.

It is a monotype genus (Sendra and Deharveng 2020).

#### Distribution.

Endemic in China (Guangxi).

### ﻿17.- Whittencampa troglobia

Taxon classificationAnimaliaDipluraCampodeidae

Sendra & Deharveng, 2020

83CD7A51-3BCD-5899-91AA-58F6743513DD

#### Description.

Body 5.2−7.0 mm length; antennae with 54–56 antennomeres; antennae 1.3−1.4 longer than body length; antennomeres more than twice longer than wide; ventral thick sensillum of the third antennomere; cuticle reticulated at high magnifications; clothing setae barbed with thin barbs: pronotum with 1+1 *ma*, 1+1 *la_4_*, 2+2 *lp_1_*_,*3*_; mesonotum with 1+1 *ma*, 1+1 *la*, 2+2 *lp_2_*_,*3*_; metanotum with 1+1 *ma*, 2+2 *lp_2_*_,*3*_; all notal macrosetae barbed with thin barbs; metathoracic legs reaching end of abdomen; legs 0.4−0.5× as long as body; cerci with seven primary articles plus the basal one; cerci 1.3 longer than body length; cercal articles covered with 2–16 whorls of thin and long macrosetae with tiny barbs on distal third, combined with whorls of smooth thin setae shorter than macrosetae.

#### Habitat.

Deep subterranean ecosystems, observed in cave habitats such as water films on speleothems.

#### Distribution.

Endemic in China (Guangxi).

#### References.

Sendra and Deharveng (2020).

### ﻿Genus Hubeicampa

Taxon classificationAnimaliaDipluraCampodeidae

Sendra & Lips, 2021

5911712C-67FC-5639-B5BC-C23C93FACDBA

#### Diagnosis.

Dense pubescence of thin micro−barbs on all kind of setae; no more than 4+4 macrosetae on pronotum, 3+3 on mesonotum and 2+2 on metanotum; one or two dorsal macrosetae on metathoracic femora and one or two on tibia; subequal elbowed claws with lateral crests; basal and ventral portion of claws covered with very small, thin, spiniform formations that look pubescent under optical microscope; laminar lateral processes of the pretarsus completely covered with dense micro–barbs; urotergites V–VII with 3+3 or 4+4 *post* macrosetae and without lateral anterior macrosetae; up to 12+12 macrosetae on first urosternite; 4+4 or 5+5 macrosetae on second to seventh urosternites; 1+1 macrosetae on eighth urosternite; first urosternite in males without glandular *g_1_* setae and appendages with glandular *a_1_* setae.

#### Remarks.

Two species have been already described (Condé 1993a; Sendra et al. 2021b).

#### Distribution.

Endemic in China (Hubei).

### ﻿18.- Hubeicampa lipsae

Taxon classificationAnimaliaDipluraCampodeidae

(Condé, 1993)

3EB03090-05FE-54F1-8A92-3225B7A4A121

#### Description.

Body length 4.0−7.1 mm; cuticle smooth under optical microscope; pretarsus including lateral processes, calcars and stylus setae with micro-barbs; antennae 1.0−1.1× longer than body length with 43−44 antennomeres; central antennomeres longer than wide; cupuliform organ with about ten globose sensilla; subcylindrical sternal sensillum of the third antennomere; pronotum with 1+1 *ma*, 1+1 *la*, 2+2 *lp*, mesonotum 1+1 *ma*, 2+2 *lp* and metanotum 1+1 *ma*, 1+1 *lp*; metathoracic leg 0.6−0.8× as long as body; two dorsal macrosetae on femur and two sternal macrosetae on tibia; lateral processes laminar covered by micro-barbed; urotergites I−IV with 1+1 *post*, V with 2+2−3+3 *post*, VI−VII with 4+4 *post*, VIII with 5+5 *post* macrosetae; male and female with subcylindrical appendages on urosternite I bearing *a_1_* glandular setae.

#### Habitat.

Subterranean ecosystems, found in cave habitats.

#### Distribution.

Endemic in China (Hubei).

#### References.

Condé (1993a); Sendra et al. (2021b).

### ﻿19.- Hubeicampa melissa

Taxon classificationAnimaliaDipluraCampodeidae

Sendra & Lips, 2021

4B092545-3CD0-550E-BA85-129799EDDADC

#### Description.

Body length 6.0−10.4 mm; cuticle reticulated observed at high magnifications; micro-barbed setae, macrosetae, stylus setae, calcars, clothing setae and marginal setae; antennae 1.2× longer that body length with 49 antennomeres; central antennomeres three times longer than wide; pronotum with 1+1 *ma*, 1+1 *la_2_*, 2+2 *lp_1_*_,*3*_, mesonotum with 1+1 *ma*, 1+1 *la*, 0−1+1 *mp*, metanotum with 1+1 *ma*; femur with one short dorsal macrosetae; ventral short tibial macrosetae; subequal elbowed claws with lateral crests covered with small thin, spiniform formations; laminar lateral process of the pretarsus completely covered by thick micro-barbed; urotergites V with 1+1 *post*, VI−VII with 3+3 *post*, VIII with 5+5 *post*, and abdominal segment IX with 6+6 *post* macrosetae; urosternite I with 12+12 macrosetae, II−VII with 5+5 macrosetae, VIII with 1+1 macrosetae; females urosternite I with coniform appendages with ***a_1_*** glandular setae; male urosternite with subcylindrical appendages with *a_1_* glandular setae; cerci 1.7× longer than body length, with six primary articles with long macrosetae a one whorl of distal setae.

#### Habitat.

Subterranean ecosystems, found in cave habitats.

#### Distribution.

Endemic in China (Hubei).

#### References.

Sendra et al. (2021b).

### ﻿Subfamily Lepidocampinae

Taxon classificationAnimaliaDipluraCampodeidae

Condé, 1956

A0D87B12-8EC6-5836-B6CC-BB56F4EA0E3B

#### Diagnosis.

Setae and macrosetae on body, thorax, and abdomen covered by scales too; pretarsus with unguiculus (long curved spine between claws); setiform or laminar lateral processes densely covered by barbs.

#### Remarks.

A total of 18 species belong to two genera *Lepidocampa* Oudemans, 1890 and *Sinocampa* Chou & Chen, 1981.

#### Distribution.

Widely distributed in the continents of the Southern Hemisphere (Condé 1956; Paclt 1957).

### ﻿Genus Lepidocampa

Taxon classificationAnimaliaDipluraCampodeidae

Oudemans, 1890

187068C5-1D8F-52BB-AAC4-4A6442B9BBC5

#### Diagnosis.

Laminar lateral processes densely covered by barbs.

#### Remarks.

It gathers 16 species already described (Condé 1956; Paclt 1957; Sendra et al. 2017).

#### Distribution.

Widely distributed in the continents of the Southern Hemisphere (Condé 1956; Paclt 1957; Sendra et al. 2017).

### ﻿20.- Lepidocampa weberi

Taxon classificationAnimaliaDipluraCampodeidae

Oudemans, 1890

FC61C832-D1DC-5CFE-A7F7-89ABBAFE5EF1

Fig. 11

#### Description.

Body 2.5−4.5 mm length; cuticle plain; antennae 0.5−0.7× as long as body with 19−27 (juveniles), 28−33 (adults) antennomeres 0.8 slightly longer than wide; large thick bacilliform tergal sensillum of the third antennomere; pronotum and mesonotum with 3+3 (*ma*, *la*, *lp*), metanotum with 2+2 (*la*, *lp*) macrosetae relatively long barbed macrosetae, *lp* the longest; short clothing setae on head, antennae, legs and cerci; substituted by scales in thorax and abdomen; metathoracic leg 0.3−0.4× as long as body with one long tergal macrosetae on femur and one ventral short macrosetae on tibia; urotergites II−III with 1+1 *lp* long barbed macrosetae; urotergites IV−VII with 3+3 *lp* long barbed macrosetae; urotergites VIII with 4+4 *lp* long barbed macrosetae; urosternite I with 6+6 barbed macrosetae; urosternite II−VII with 3+3 barbed macrosetae; cerci 0.5−0.6× as long as body with eight articles plus basal one, covered with long barbed macrosetae, plus a few clothing setae.

**Figure 11.**
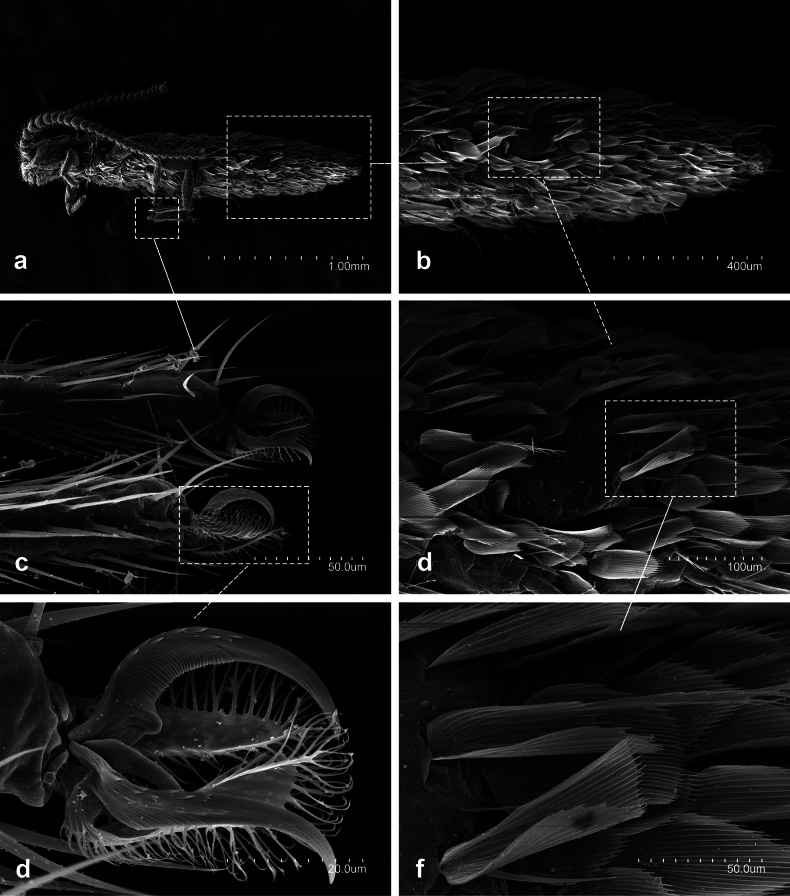
*Lepidocampaweberi* Oudemans, 1890 **a** habitus **b** distal portion of abdomen **c** distal portion metathoracic tarsus and their pretarsus **d** detail of abdomen setae **e** metathoracic pretarsus **f** detail of abdominal scales.

#### Habitat.

Upper to lower layers in forest soils.

#### Distribution.

Ethiopian and Oriental regions, including some boundaries in the northeast of Oriental region, China included (Guangdong, Hong Kong, Macao, Fujian, Hubei, Hunan, Yunnan, Shanghai, Jiangsu, Anhui, Zhejiang, Jiangxi, Sichuan, Guizhou, Guangxi, and Hainan).

#### References.

Bareth (1992); Bareth and Condé (1974); Condé (1953, 1954, 1955, 1958, 1960, 1982, 1989, 1990, 1992, 1993b); Condé and Jacquemin−Nguyen (1968); Silvestri (1931, 1933b); Xie (2000); Xie and Yang (1992, 1993).

### ﻿21.- Lepidocampa polettii

Taxon classificationAnimaliaDipluraCampodeidae

Silvestri, 1931

DDD7414A-2B1F-5453-9286-4C3D39D758E5

#### Description.

Body 1.5−2.9 mm length; cuticle no describe; antennae 0.4× as long as body with 16−19 antennomeres; sensillum of the third antennomere unknown; pronotum and mesonotum with 3+3 (*ma*, *la*, *lp*), 2+2 (*la*, *lp*) and 1+1 (*lp*) relatively long barbed macrosetae; short clothing setae on head, antennae, legs and cerci; substituted mostly by scales in thorax and abdomen; metathoracic leg 0.4× as long as body: without dorsal femoral macrosetae: one ventral macrosetae on tibia; urotergites II-III with 1+1 *lp* long barbed macrosetae; urotergites IV−VII with 3+3 *lp* long barbed macrosetae; urosternite I with 6+6 barbed macrosetae; urosternite II−VII with 4+4 barbed macrosetae; cerci 0.6× as long as body with ten articles plus basal one, covered with numerous setae.

#### Habitat.

Soil.

#### Distribution.

Southeast Asia, in China (Fujian and Hubei) and Vietnam.

#### References.

Silvestri (1931).

### ﻿22.- Lepidocampa takahashii

Taxon classificationAnimaliaDipluraCampodeidae

Silvestri, 1931

C415B95D-D903-5455-BF52-FF1EEB9C78D5

#### Description.

Body 2.9 mm length; cuticle no describe; antennae 0.4× as long as body with 17 antennomeres; sensillum of the third antennomere unknown; pronotum and mesonotum with 3+3 (*ma*, *la*, *lp*), 2+2 (*la*, *lp*) and 1+1 (*lp*) relatively long barbed macrosetae; short clothing setae on head, antennae, legs and cerci; substituted mostly by scales in thorax and abdomen; metathoracic leg 0.4× as long as body without tergal macrosetae on femur and no ventral macrosetae on tibia; urotergites III with 1+1 *lp* macrosetae; urotergites IV with 2+2 *lp* macrosetae; urotergites V−VII with 3+3 *lp* macrosetae; urotergite VIII and abdominal segment IX with 4+4 *lp* long barbed macrosetae; all tergal macrosetae barbed and long;-urosternite I with 6+6 barbed macrosetae; urosternites II-VII with 9+9 barbed macrosetae.

#### Habitat.

Soil.

#### Distribution.

Endemic in China (Yunnan, Sichuan, Guangdong, and Guangxi).

#### References.

Silvestri (1931).

### ﻿Genus Sinocampa

Taxon classificationAnimaliaDipluraCampodeidae

Chou & Chen, 1981

1D41831E-D818-5D0D-A7E2-ED82EF2176ED

#### Diagnosis.

Setiform lateral processes densely covered by long barbs giving a feather-like form.

#### Remarks.

A genus with two exclusive species (Chou and Chen 1981).

#### Distribution.

Endemic in China, described from Sichuan (Chou and Tong 1981).

### ﻿23.- Sinocampa huangi

Taxon classificationAnimaliaDipluraCampodeidae

Chou & Chen, 1981

C8A44728-1A12-5CEE-8569-C9BE290DCF87

#### Description.

Antennae with 28−33 moniliform antennomeres; pronotum with 6+6, mesonotum with 7+7 and metanotum with 8+8 relatively long barbed macrosetae; two macrosetae on femur and one ventral macrosetae on tibia of the third pair of legs; urotergites I no macrosetae, urotergites II-III with 1+1 *lp* long barbed macrosetae; urotergites IV-VI with 3+3 *lp* long barbed macrosetae; urosternite I with 4+4 barbed macrosetae; urosternite VIII with 1+1 barbed macrosetae; setae stylus with a few barbs; cerci with long barbed macrosetae, plus a few clothing setae; appendages urosternite I subcylindrical; males with a large field of setae on the posterior half portion of first urosternite.

#### Habitat.

Soil.

#### Distribution.

Endemic in China (Xizang and Sichuan).

#### References.

Chou and Chen (1981).

### ﻿24.- Sinocampa zayuensis

Taxon classificationAnimaliaDipluraCampodeidae

Chou & Chen, 1981

DDE475FD-7BF3-5489-A001-0444BBCB749D

#### Description.

Pronotum with 5+6, mesonotum and metanotum with 8+7 relatively long barbed macrosetae; without dorsal femoral and ventral tibial macrosetae; urosternite I with 6+6; urosternites II−VII with 3+3 and urosternite VIII with 1+1 barbed macrosetae; setae stylus with a few barbs; cerci with numerous short clothing setae; appendages urosternite I subcylindrical; males with a large field of setae on the posterior half portion of first urosternite.

#### Habitat.

Soil.

#### Distribution.

Endemic in China (Xizang and Sichuan).

#### References.

Chou and Chen (1981).

### ﻿Subfamily Syncampinae

Taxon classificationAnimaliaDipluraCampodeidae

Paclt, 1957

1EAAC5C3-70B5-547A-BDFC-38AF5EDE2350

#### Diagnosis.

Short clothing setae on head, antennae, legs, and cerci; substituted mostly by scales in thorax and abdomen; with one and one long tergal macrosetae on femur and one ventral short macrosetae on tibia; pretarsus without unguiculus (long curved spine between claws); laminar lateral processes with ventral part covered by barbs; claws with a small lateral external prolongation.

#### Remarks.

A family with a single genus and species (Paclt 1957).

#### Distribution.

Endemic in China (Silvestri 1931).

### ﻿Genus Syncampa

Taxon classificationAnimaliaDipluraCampodeidae

Silvestri, 1931

4BF5D9DC-B53F-57C1-8923-CEDC35AB2980

#### Diagnosis.

Pretarsus without unguiculus; laminar lateral processes ending round with ventral part covered by barbs; claws with a small lateral external prolongation.

#### Remarks.

A monotypic genus (Silvestri 1931).

#### Distribution.

Endemic in China (Silvestri 1931).

### ﻿25.- Syncampa smithii

Taxon classificationAnimaliaDipluraCampodeidae

Silvestri, 1931

F22F3CA5-273E-55B0-A743-6A1FE1CD2D0C

#### Description.

Body 5.0 mm length; epicuticle no describe; antennae 0.8× as long as body with 35 antennomeres as long as wide; unknown third antennomere sensillum; pronotum with 3+3 (*ma*, *la*, *lp*), mesonotum with 2+2 (*la*, *lp*) and metanotum with 1+1 (*lp*) relatively long barbed macrosetae; metathoracic leg 0.5× as long as body; urotergites II−III with 1+1 *post*, urotergites IV−VII with 3+3 *lp*, urotergites VIII 4+4 *post* barbed macrosetae; urosternite I with 6+6 and urosternite II−VII with 4+4 barbed macrosetae.

#### Habitat.

Deep layers in soil, probably an endogean species.

#### Distribution.

Endemic in China (Guangdong).

#### References.

Silvestri (1931).

### ﻿Taxonomic key to Chinese campodeids

**Table:** 

1	Body covered by normal setae and macrosetae	**2**
–	Body covered by setae modified in scales in addition to normal setae and macrosetae	**3**
2	Pronotal macrosetae formula none or 1+1 medial anterior, 1+1 lateral anterior, and 1+1 lateral posterior. Campodeinae Condé, 1956	**4**
–	The pronotum never has < 4+4 macrosetae, usually at least 1+1 medial anterior, 1+1 lateral anterior, and 2+2 lateral posterior. Plusiocampinae Paclt, 1957	**11**
3	Setae and macrosetae on body, thorax, and abdomen covered by scales; pretarsus with unguiculus (long curved spine between claws); setiform or laminar lateral processes densely covered by barbs. Lepidocampinae Condé, 1956	**16**
–	Pretarsus without unguiculus (long curved spine between claws); claws with a small lateral external prolongation; laminar lateral processes with ventral part covered by barbs	**Syncampinae Paclt, 1957: *Syncampa* Silvestri, 1931: *Syncampasmithii* Silvestri, 1931**
4	Simple, smooth, subequal, curved claws with smooth, setiform, pretarsus processes. ***Campodea*** Westwood, 1842	**5**
–	Subequal slightly curved claws with a small latero-ventral spine without lateral pretarsus processes. *Metriocampa* Silvestri, 1912	**7**
–	Subequal slightly curved claws with small lateral crests and setiform lateral process with barbs	***Leniwytsmania* Paclt, 1957: *Leniwytsmaniaorientalis* (Silvestri, 1931)**
–	Subequal elbow-like claws; laminar lateral process ending with barbs	***Pseudolibanocampa* Xie & Yang, 1991: *Pseudolibanocampasinensis* Xie & Yang, 1991**
–	Subequal elbowed claws with ridges on dorsal side that look like very small lateral crests under optical microscope; without lateral processes	***Pacificampa* Chevrizov, 1978: *Pacificampawudonghuii* Sendra, 2021**
5	Pronotum, mesonotum, and metanotum with 3+3 (medial anterior, lateral anterior, and lateral posterior), 3+3 (medial anterior, lateral anterior, and lateral posterior), 2+2 (medial anterior and lateral posterior) macrosetae	**6**
–	Pronotum with 2+2 (medial anterior and lateral posterior) short thick macrosetae	***Campodea* (?) *pagei* Silvestri, 1931**
6	Urotergite IV with 1+1 lateral anterior and 1+1 lateral posterior macrosetae	**Campodea (Campodea) mondainii Silvestri, 19317**
–	Urotergite IV with 1+1 lateral posterior macrosetae	**Campodea (Campodea) ishii Silvestri, 1931**
7	Pronotum with 3+3 (medial anterior, lateral anterior and lateral posterior), mesonotum with 2+2 (medial anterior, lateral anterior), and metanotum with 1+1 (medial anterior) macrosetae	***Metriocampaurumqiensis* Chou & Chen, 1991**
–	Fewer notal macrosetae formula of 3+3, 2+2, and 1+1 macrosetae	**8**
8	Pronotum with 2+2 (medial anterior and lateral posterior) mesonotum with 2+2 (medial anterior, lateral anterior), and metanotum with 1+1 (medial anterior) macrosetae	**9**
–	Pronotum with 2+2 (medial anterior and lateral posterior), mesonotum with 1+1 (medial anterior) macrosetae	**10**
–	Pronotum with 2+2 (medial anterior and lateral posterior) macrosetae	***Metriocampapackardi* Silvestri, 1912**
9	1+1 lateral posterior macrosetae on urotergite VIII	***Metriocampakuwayamai* Silvestri, 1931**
–	2+2 lateral posterior macrosetae on urotergite VIII	***Metriocampawyyanlienis* Xie & Yang, 1991**
10	Thin and short clothing setae	***Metriocampamatsumurae* Silvestri, 1931**
–	Thick and short clothing setae	***Metriocampasahi* Silvestri, 1931**
11	Subequal elbow-like claws with lateral crests; setiform lateral processes	***Plusiocampa* Silvestri, 1912: *Plusiocampasinensis* Silvestri, 1931**
–	Laminar setiform processes	**12**
12	Unequal elbowed claws with lateral crests without lateral processes	***Plutocampa* Chevrizov, 1978: *Plutocampamethoria* Chevrizov, 1978**
–	Unequal elbowed claws with lateral crests with lateral processes barbed	**13**
13	Setiform and barbed lateral processes	***Whittencampa* Sendra & Deharveng, 2020: *Whittencampatroglobia* Sendra & Deharveng, 2020**
–	Barbed and laminar lateral processes	**14**
14	Laminar lateral processes with long barbs	***Cestocampa* Condé, 1956: *Cestocampakashiensis* Chou & Chen, 1980**
–	Laminar pretarsus lateral processes with long broad fringes as result of their fragmentation	***Anisuracampa* Xie & Yang, 1991: *Anisuracampasuoxiensis* Xie & Yang, 1991**
–	Laminar lateral processes of the pretarsus completely covered with dense micro-barbs. *Hubeicampa* Sendra & Lips, 2021	**15**
15	Mesonotum with 1+1 medial anterior, 2+2 lateral posterior and metanotum, 1+1 medial anterior, 1+1 lateral posterior macrosetae; two dorsal macrosetae on femur ……………	***Hubeicampalipsae* (Condé, 1993)**
–	Mesonotum with 1+1 medial anterior, 1+1 lateral anterior, 0-1+1 medial posterior, metanotum with 1+1 medial anterior macroseta; femur with one short dorsal macrosetae	***Hubeicampamelissa* Sendra & Lips, 2021**
16	Laminar lateral processes densely covered by barbs. ***Lepidocampa*** Oudemans, 1890	**17**
–	Setiform lateral processes densely covered by long barbs giving a feather-like form. *Sinocampa* Chou & Chen, 1981	**19**
17	Mesonotum 2+2 lateral anterior and lateral posterior macrosetae, metanotum with 1+1 lateral posterior macroseta; without dorsal femoral macrosetae	**18**
–	Mesonotum with 3+3 (medial anterior, lateral anterior, and lateral posterior) macrosetae; metanotum with 2+2 (lateral anterior, lateral posterior) macrosetae; one dorsal femoral macrosetae	***Lepidocampaweberi* Oudemans, 1890**
18	Urotergite IV with 3+3 lateral posterior long barbed macrosetae	***Lepidocampapolettii* Silvestri, 1931**
–	Urotergite IV without macrosetae	***Lepidocampatakahashii* Silvestri, 1931**
19	Two macrosetae on femur	***Sinocampahuangi* Chou & Chen, 1981**
–	Without femoral macrosetae	***Sinocampazayuensis* Chou & Chen, 1981**

## ﻿Discussion and conclusions

Campodeids is a well-represented and diverse family among the basal group of diplurans (Condé 1956; Paclt 1957; Sendra et al. 2021a). Campodeidae has 491 species described from all continents except in Antarctica, since they never managed to overpass the Polar circles, and 50% of the species are known from West Palearctic biogeographical region of which approximately 50 species are found in East Asia (Sendra et al. 2021b). The diversity of Campodeidae in China is represented by 25 species belonging to four subfamilies, Campodeinae, Plusiocampinae, Lepidocampinae and Syncampinae, the last of which is endemic in China (Table 1).

**Table 1.** Campodeidae diversity in China.

Subfamily	Genus	Species	Ecology		Distribution					
			Soil-dweller	Cave-adapted	Endemic in China	East Asia	Holarctic	Oriental/ Ethiopian	Oriental	Worldwide
**Campodeinae** Condé, 1956	**Campodea** Westwood, 1842		**x**	**x**						**x**
		**Campodea (Campodea) mondainii** Silvestri, 1931	**x**			**x**				
		**Campodea (Campodea) ishii** Silvestri, 1931	**x**			**x**				
		***Campodea* (?) *pagei*** Silvestri, 1931	**x**		**x**					
	**Metriocampa** Silvestri, 1912		**x**				**x**			
		***Metriocampakuwayamai*** Silvestri, 1931	**x**			**x**				
		***Metriocampamatsumurae*** Silvestri, 1931	**x**			**x**				
		***Metriocampapackardi*** Silvestri, 1912	**x**				**x**			
		***Metriocampasahi*** Silvestri, 1931	**x**		**x**					
		***Metriocampaurumqiensis*** Chou & Chen, 1980	**x**		**x**					
		***Metriocampawuyanlinensis*** Xie & Yang, 1991	**x**		**x**					
	***Leniwytsmania*** Paclt, 1957		**x**		**x**					
		***Leniwytsmaniaorientalis*** (Silvestri, 1931)	**x**		**x**					
	***Pseudolibanocampa*** Xie & Yang, 1991		**x**		**x**					
		***Pseudolibanocampasinensis*** Xie & Yang, 1991	**x**		**x**					
	***Pacificampa*** Chevrizov, 1978			**x**		**x**				
		***Pacificampawudonghuii*** Sendra, 2021		**x**	**x**					
**Plusiocampinae** Paclt, 1957	***Plusiocampa*** Silvestri, 1912		**x**	**x**			**x**			
		***Plusiocampa* (incer. sed.) *sinensis*** Silvestri, 1931	**x**		**x**					
	***Cestocampa*** Silvestri, 1912		**x**	**x**			**x**			
		***Cestocampakashiensis*** Chou & Chen, 1980	**x**		**x**					
	***Plutocampa*** Chevrizov, 1978		**x**			**x**				
		***Plutocampamethoria*** Chevrizov, 1978	**x**			**x**				
	***Anisuracampa*** Xie & Yang, 1991		**x**	**x**					**x**	
		***Anisuracampasuoxiensis*** Xie & Yang, 1991	**x**		**x**					
	***Whittencampa*** Sendra & Deharveng, 2020			**x**	**x**					
		***Whittencampatroglobia*** Sendra & Deharveng, 2020		**x**	**x**					
	***Hubeicampa*** Sendra & Lips, 2021			**x**	**x**					
		***Hubeicampalipsae*** (Condé, 1993)		**x**	**x**					
		***Hubeicampamelissa*** Sendra & Lips, 2021		**x**	**x**					
**Lepidocampinae** Condé, 1956	***Lepidocampa*** Oudemans, 1890		**x**	**x**				**x**		
		***Lepidocampaweberi*** Oudemans, 1890	**x**					**x**		
		***Lepidocampapolettii*** Silvestri, 1931	**x**			**x**				
		***Lepidocampatakahashii*** Silvestri, 1931	**x**		**x**					
	***Sinocampa*** Chou & Tong, 1981		**x**		**x**					
		***Sinocampahuangi*** Chou & Chen, 1981	**x**		**x**					
		***Sinocampazayuensis*** Chou & Chen, 1981	**x**		**x**					
**Syncampinae** Paclt, 1957	***Syncampa*** Silvestri, 1931		**x**		**x**					
		***Syncampasmithii*** Silvestri, 1931	**x**		**x**					

The most diverse of the Campodeidae subfamilies in China is Campodeinae with 12 species: 11 of these are soil-dwellers with wide distribution areas, belonging to the genera *Campodea* (3 species), *Metriocampa* (6 species), *Leniwytsmania* (1 species), and *Pseudolibanocampa* (1 species), and one cave-adapted species belonging *Pacificampa*. Among them, *Leniwytsmania* and *Pseudolibanocampa* are monospecific and endemic in China. The second subfamily is Plusiocampinae with seven species. Of these, four soil-dwelling species belong to *Plusiocampa* (1 species), *Cestocampa* (1 species), *Anisuracampa* (1 species), and *Plutocampa* (1 species). *Anisuracampa* and *Plutocampa* are endemic in East Asia and *Plusiocampa* and *Cestocampa* are widely distributed in the Western Palearctic region. The other three species are cave-adapted species in several karstic areas, belonging to two endemic genera in China: *Hubeicampa* (2 species) and *Whittencampa* (1 species). The third subfamily is Lepidocampinae, known by five soil-dwelling species belonging to *Lepidocampa* (3 species) and *Sinocampa* (2 species) genera. *Sinocampa* is endemic in China, and one *Lepidocampa* species is also endemic. Finally, Syncampinae is a monogeneric and monospecific subfamily endemic in China (Table 1).

Based on the collection data so far, the southeast region of China, which has a larger population and a more developed economy, hosts a higher number of Campodeidae species. This is partly due to the more extensive surveys conducted in these areas, as well as the region’s warmer climate and lower altitudes, which support greater dipluran species diversity compared to the colder northern and high-altitude western regions. Among the 25 known Campodeidae species distributed in China, *Lepidocampaweberi* and *Campodeamondainii* are widely distributed, occurring in more than ten administrative divisions. *Metriocampaurumqiensis* and *Cestocampakashiensis* are restricted to the northwest, while *Leniwytsmaniaorientalis*, *Pseudolibanocampasinensis*, and *Lepidocampatakahashii* are confined to the southwest. Three species, *Campodeapagei*, *Lepidocampapolettii*, and *Syncampasmithii*, have not been rediscovered since Silvestri’s (1931) report. The distribution of the five cave-dwelling species, *Pacificampawudonghuii*, *Plutocampamethoria*, *Whittencampatroglobia*, *Hubeicampalipsae*, and *Hubeicampamelissa*, is very unique, with each species found exclusively in a single cave (Table 1).

The diversity of Chinese campodeid species is higher when compared to other areas within the Eastern Palearctic and East Asia but is lower than the diversity in the Western Palearctic, especially in the Euro-Mediterranean region, due to the more sampling efforts (Sendra et al. 2020a, b). Nevertheless, East Asia has emerged as an origin centre for at least the Plusiocampinae, Lepidocampinae, and Syncampinae subfamilies (Condé 1956; Sendra et al. 2021a, b, 2022).

## Supplementary Material

XML Treatment for Campodeinae

XML Treatment for Campodea

XML Treatment for Campodea

XML Treatment for Campodea (Campodea) mondainii

XML Treatment for Campodea (Campodea) ishii

XML Treatment for Campodea (?) pagei

XML Treatment for Metriocampa

XML Treatment for Metriocampa kuwayamai

XML Treatment for Metriocampa matsumurae

XML Treatment for Metriocampa packardi

XML Treatment for Metriocampa sahi

XML Treatment for Metriocampa urumqiensis

XML Treatment for Metriocampa wuyanlinensis

XML Treatment for Leniwytsmania

XML Treatment for Leniwytsmania orientalis

XML Treatment for Pseudolibanocampa

XML Treatment for Pseudolibanocampa sinensis

XML Treatment for Pacificampa

XML Treatment for Pacificampa wudonghuii

XML Treatment for Plusiocampinae

XML Treatment for Plusiocampa

XML Treatment for Plusiocampa

XML Treatment for Cestocampa

XML Treatment for Cestocampa kashiensis

XML Treatment for Plutocampa

XML Treatment for Plutocampa methoria

XML Treatment for Anisuracampa

XML Treatment for Anisuracampa suoxiensis

XML Treatment for Whittencampa

XML Treatment for Whittencampa troglobia

XML Treatment for Hubeicampa

XML Treatment for Hubeicampa lipsae

XML Treatment for Hubeicampa melissa

XML Treatment for Lepidocampinae

XML Treatment for Lepidocampa

XML Treatment for Lepidocampa weberi

XML Treatment for Lepidocampa polettii

XML Treatment for Lepidocampa takahashii

XML Treatment for Sinocampa

XML Treatment for Sinocampa huangi

XML Treatment for Sinocampa zayuensis

XML Treatment for Syncampinae

XML Treatment for Syncampa

XML Treatment for Syncampa smithii

## ﻿Additional information

### Author ORCIDs

Yun Bu https://orcid.org/0000-0002-7177-9686

Yun-Xia Luan https://orcid.org/0000-0003-3573-7144

### Data availability

All of the data that support the findings of this study are available in the main text.

## References

[B1] Bareth C (1992) Diploures Campodéidés du Nord-Est de Madagascar (InsectaApterygota). Journal of African Zoology 106(6): 489−497. https://pascal-francis.inist.fr/vibad/index.php?action=getRecordDetail&idt=4560919

[B2] Bareth C Condé B (1958) Campodéidés endogés de l’ouest des États-Unis (Washinton, Oregon, Californie, Arizona). Bulletin de la Société Linneenne de Lyon 8: 226−248; 9: 265−276; 10: 297−304. 10.3406/linly.1958.8012

[B3] Bareth C Condé B (1974) Diploures Campodéidés des Iles Salomon. Revue d’Ecologie et de Biologie du Sol IX (2): 235−256.

[B4] Chevrizov BP (1978) Two new genera of the Family Campodeidae from the Far East Caves. Zoologichesky Zhurnal 57 (2): 197−205. https://digitalcommons.usf.edu/kip_articles/6532/

[B5] Chou I Chen T (1980) Two new species of Campodeidae from Xinjiang (Apterygota: Diplura). Entomotaxonomia II (2): 157−160. http://xbkcflxb.alljournal.net/xbkcflxb/ch/reader/view_abstract.aspx?file_no=19800233&flag=1

[B6] Chou I Chen T (1981) Diplura: Campodeidae. In: the Comprehensive Scientific Expedition Team of the Tibetan Plateau, Chinese Academy of Sciences (Eds) Insects of Xizang (The Series of the Comprehensive Scientific Expedition to the Qinghai-Xizang Plateau, Volume 1). Science Press, Pecking, 47−52.

[B7] Condé B (1953) Campodéidés de Madagascar et de l’Ili de la Reunion. Mémoires de l’Institut Scientifique de Madagascar Série E, IV: 617−637.

[B8] Condé B (1954) Sur la présence de Lepidocampa (L.) weberi Oudemans en Afrique (DipluraCampodeidae). Annales du Musée de Congo, Tervuren, in-4, Zoologie 1: 332−333.

[B9] Condé B (1955) Contribution à l’étude de la faune entomologique du Ruanda-Urundi (Mission P. Basilewsky 1953). XLVII. DipluraCampodeidae. Annales du Musée du Congo Tervuren in-8 Zoologie 40: 9.

[B10] Condé B (1956) Matériaux pour une Monographie des Diploures Campodéidés. Mémoires du Muséum National d’Histoire naturelle Série A Zoologie 12: 1–202.

[B11] Condé B (1958) British Museum Nepal Expedition 1954: Protoures et Diploures Campodéidés. Proceedings of the Royal Entomological Society of London (B) 27 (11–12): 189−194. 10.1111/j.1365-3113.1958.tb00405.x

[B12] Condé B (1960) Mission zoologique de l’IRSAC en Afrique orientale (P. Basilewsky et N. Leleup, 1957). XXXVII DipluraCampodeidae. Annales du Musée du Congo Tervuren in-8 Zoologie 88: 9−13.

[B13] Condé B (1982) Diploures Campodéidés de Papouasie. Revue suisse Zoologie 89(3): 731−748. 10.5962/bhl.part.82471

[B14] Condé B (1989) Prodromes d’une evolution souterraine dans le genre *Lepidocampa* Oudemans (DipluraCampodeidae). Mémoires de Biospéologie XVI: 153−156.

[B15] Condé B (1990) Diploures Campodéidés de Bornéo. Revue suisse Zoologie 97(2): 465−475. 10.5962/bhl.part.79749

[B16] Condé B (1992) Campodéidés des grottes des Célebes (Insectes, Diploures). Mémoires Biospéologie XIX: 155−158.

[B17] Condé B (1993a) Premiers Campodeidae cavernicoles de China, comme exemple de l’évolution souterraine de la Familie (Diplura). Revue suisse Zoologie 100 (4): 823−828. 10.5962/bhl.part.79886

[B18] Condé B (1993b) Campodeidae de Sumatra et de Singapour (Diplura). Revue suisse Zoologie 100(4): 949−959. 10.5962/bhl.part.79894

[B19] Condé B Geeraert P (1962) Campodéidés endogés du centre des Éstats-Units. Archives de Zoologie expérimentale et générale 101(3): 73−16.

[B20] Condé B Jacquemin-Nguyen M (1968) Diplopodes Pénicillates et Diploures Campodéidés. Khumbu Himal 3(1): 4−8.

[B21] Condé B Thomas J (1957) Contribution à la faune des Campodéidés de Californie. Bulletin de la Société Linneenne de Lyon 4: 81−96, 5: 118−127, 6: 142−155. 10.3406/linly.1957.7893

[B22] Denis JR (1949) Ordre des Diploures. In: Grassé PP (Ed.) Traité de Zoologie IX.Masson, Paris, 160–185.

[B23] Ferguson LM (1997) A report on a new species of *Pacificampa* (Diplura: Campodeidae) from a cave in China and comparison of some North American genera to *Pacificampa* and *Plutocampa* previously only known from the Far East of Russia. Proceedings of the 12^th^ International Congress of Speleology, La Chaux-de-Fonds, Neuchâtel, Swizerland 3: 315−317.

[B24] Koch M (2001) Diplura. In: Resh VH Cardé RT (Eds) Encyclopedia of Insects.2 edn. Elsevier, Amsterdam, 281–283. 10.1016/B978-0-12-374144-8.00084-9

[B25] Kukalová-Peck J (1987) New Carboniferous Diplura, Monura, and Thysanura, the hexapod ground plan, and the role of thoracic side lobes in the origin of wings (Insecta). Canadian Journal of Zoology 65: 2327–45. 10.1139/z87-352

[B26] Paclt J (1957) Diplura. In: Genera. Wytsman P, Insectorum 212º fasc: 1−123.

[B27] Pagés J (1959) Remarques sur la classification des diploures. Travaux du Laboratoire de Zoologie et de la Station Aquicole Grimaldi de la Faculte des Sciences de Dijon 26: 1–25.

[B28] Pagés J (1989) Sclérites et appendices de l’abdomen des Diploures (Insecta, Apterygota). Archives des Sciences Genève 42: 509–551.

[B29] Rusek J (1982) *Octostigmaherbivora* gen. & sp. (Diplura: Projapygidae: Octostigmatidae n. fam.) injuring plant roots in the Tonga Islands. New Zealand Journal Zoology 9: 25–32. 10.1080/03014223.1982.10423833

[B30] Sánchez-García A Sendra A Davis S Grimaldi DA (2023a) Fossil diversity in ‘dawn’ hexapods (Diplura: Projapygoidea), with direct evidence for being chemically predaceous in the Cretaceous. Zoological Journal of the Linnean Society 198, 3: 847–870. 10.1093/zoolinnean/zlac101

[B31] Sánchez-García A Sendra A David SR Grimaldi DA (2023b) ‘Dawn’ hexapod in Cenozoic ambers (Diplura: Campodeidae). Zoological Journal of the Linnean Society XX: 1–23. 10.1093/zoolinnean/zlad118

[B32] Sendra A (2015) Clase Entognatha. Orden Diplura. Revista IDE 35: 1–11.

[B33] Sendra A (2021) Chapter 8, Classe des Diplura (Diploures). In Henri-Pierre Aberlenc. Les Insectes du Monde, Museo Éd-Quæ éd.: 213−217.

[B34] Sendra A Deharveng L (2020) *Whittencampatroglobia*, a new genus and species of troglomorphic Plusiocampinae from China (Diplura: Campodeidae). Raffles Bulletin of Zoology Supplement 35: 68–77. 10.26107/RBZ-2020-0039

[B35] Sendra A Arnedo MA Ribera C Teruel S Bidegaray-Batista L Condé B (2012) Revision of *Cestocampa* Condé (Diplura, Campodeidae), with description of a new species from caves in the eastern Iberian Peninsula. Zootaxa 3242: 43−56. 10.11646/zootaxa.3252.1.2

[B36] Sendra A Jiménez-Valverde A Rochatc J Legrosc V Gasnierc S Cazanove G (2017) A new and remarkable troglobitic *Lepidocampa* Oudemans, 1890 species from La Réunion Island, with a discussion on troglobiomorphic adaptations in campodeids (Diplura). Zoologischer Anzeiger 266: 95–104. 10.1016/j.jcz.2016.11.005

[B37] Sendra A Antić D Barranco P Borko Š Christian E Delić T Fadrique F Faille A Galli L Gasparo F Georgiev D Giachino PM Kováč L Lukić M Marcia P Miculinić K Nicolosi G Palero F Paragamian K Pérez T Polak S Prieto CE Turbanov I Vailati D Reboleira ASPS (2020a) Flourishing in subterranean ecosystems: Euro-Mediterranean Plusiocampinae and tachycampoids (Diplura, Campodeidae). European Journal of Taxonomy 591: 1–138. 10.5852/ejt.2020.728.1181

[B38] Sendra A Jiménez-Valverde A Selfa J Reboleira ASPS (2021a) Diversity, ecology, distribution and biogeography of Diplura. Insect Conserv Divers 14: 415−425. 10.1111/icad.12480

[B39] Sendra A Komerički A Lips J Luan Y Selfa J Jiménez-Valverde A (2021b) Asian cave-adapted diplurans, with the description of two new genera and four new species (Arthropoda, Hexapoda, Entognatha). European Journal of Taxonomy 731: 1–46. 10.5852/ejt.2021.731.1199

[B40] Sendra A Palero F Sánchez-García A Selfa J Sadreddin Tusun S Satar A (2022) New evidence for an Anatolian bridge: Colonization of Euromediterranean lands by cave-adapted Plusiocampinae (Diplura, Campodeidae), with establishment of a new genus. Zoologischer Anzeiger 301: 205−214. 10.1016/j.jcz.2022.10.006

[B41] Silvestri F (1911) Nuovi generi e nuove specie di Campodeidae (Thysanura) dell’America settentrionale. Bolletino del Laboratorio di Zoologia generale e agraria in Portici VI: 5−25.

[B42] Silvestri F (1931) Campodeidae (InsectaThysanura) dell’estremo Oriente. Bolletino del Laboratorio d’Entomologia agraria in Portici XXV: 286−320.

[B43] Silvestri F (1933a) Quarto contributo alla conoscenza dei Campodeidae (Thysanura) del Nord America. Bolletino del Laboratorio di Zoologia generale e agraria in Portici XXVII: 156−204.

[B44] Silvestri F (1933b) First contribution to the knowledge of the Indo-Malayan Campodeidae (Thysanura-Entotropha). Records of Indian Museum XXXV: 379−392. 10.26515/rzsi/v35/i4/1933/162581

[B45] Wang YH Huang DY Cai CY (2023) A new genus of japygids (Diplura: Japygidae) in mid-Cretaceous amber from northern Myanmar. Zootaxa 5396(1): 64–73. 10.11646/zootaxa.5396.1.12 38220980

[B46] Xie RG Yang Y (1991) Description of two new genera and three new species of Campodeidae in China (Diplura). Contributions from Shanghai Institute of Entomology 10: 95−102.

[B47] Xie RD Yang Y (1992) Identification of soil animals. 10. Arthropoda 3. Insecta, Diplura. In: Yin, Wenying Eds. Subtropical soil animals of China. Science Press. Beijing: 457−473.

[B48] Xie RD Yang Y (1993) Diplura. In: Huang, Fusheng (Eds) Insects of Wuling Mountains area, southwestern China. Science Press, Beijing, 29−30.

[B49] Xie RD (2000) Diplura of Yunnan, Southwest China. In: Aoki J, Yin WY, Imadaté G. Taxonomical Studies on the Soil Fauna of Yunnan Province in Southwest China. Tokai University Press. Tokyo.

[B50] Yin WY (2000) Pictorial keys to soil animals of China. Science Press, Beijing, New York, 727 pp.

